# Genetic predisposition to coffee consumption and the association with the early risk of atherosclerosis

**DOI:** 10.1038/s41598-026-44122-2

**Published:** 2026-03-22

**Authors:** Xiangyu Qiao, Vanessa William Toma, Jing Wang, Ángel Herraiz-Adillo, Simon Söderholm, Daniel Berglind, Susanna Calling, Bledar Daka, Mats Martinell, Frida Bergman, Pontus Henriksson, Bijar Ghafouri, Martin Ulander, Carl Johan Östgren, Claudio Cantù, Wen Zhong, Fredrik Iredahl

**Affiliations:** 1 Science for Life Laboratory, Department of Biomedical and Clinical Sciences, Division of Cell and Neurobiology, Faculty of Medicine and Health Sciences, Linköping University, Linköping, Sweden; 2 Department of Health, Medicine and Caring Sciences, Linköping University, Linköping, Sweden; 3 Department of Biomedical and Clinical Sciences, Division of Molecular and Virology, Faculty of Medicine and Health Sciences, Linköping University, Linköping, Sweden; 4 Wallenberg Centre for Molecular Medicine, Linköping University, Linköping, Sweden; 5 Centre for Epidemiology and Community Medicine, Region Stockholm, Stockholm, Sweden; 6 Center for Wellbeing, Welfare and Happiness, Stockholm School of Economics, Stockholm, Sweden; 7 Center for Primary Health Care Research, Department of Clinical Sciences Malmö, Lund University, Malmö, Sweden; 8 Office for Primary Care, Skåne University Hospital, Lund, Sweden; 9 Family Medicine, School of Public Health and Community Medicine, Institute of Medicine, Sahlgrenska Academy, University of Gothenburg, Gothenburg, Sweden; 10 Department of Public Health and Caring Sciences, Uppsala University, Uppsala, Sweden; 11 Department of Public Health and Clinical Medicine, Umeå University, Umeå, Sweden; 12 Anaesthetics, Operations and Speciality Surgery Center, Department of Clinical Neurophysiology, Region Östergötland, Linköping, Sweden; 13 Primary Health Care Center, Department of Health, Medicine and Caring Sciences, Faculty of Medicine and Health Sciences, Linköping University, Linköping, Sweden; 14 Centre of Medical Image Science and Visualization, Linköping University, Linköping, Sweden; 15 Department of Neuroscience, Karolinska Institute, Stockholm, Sweden

**Keywords:** Coffee consumption, Atherosclerosis, Multi-omics, Mendelian randomization, Biomarkers, Cardiology, Diseases, Genetics, Medical research, Risk factors

## Abstract

The cardiovascular effects of coffee consumption remain debated, particularly regarding early-stage subclinical atherosclerosis. This study investigated the association between coffee intake, genetic predisposition, and the risk of subclinical coronary and carotid atherosclerosis in 24,835 participants from the Swedish CArdioPulmonary bioImage Study (SCAPIS). Coffee intake was assessed via self-reported questionnaires. Atherosclerosis was assessed via segment involvement score (SIS), coronary artery calcium score (CACS) and carotid plaque. Observational analysis showed no significant association between coffee consumption and SIS, CACS, or carotid plaques. However, both one-sample and two-sample (SCAPIS and UK Biobank) Mendelian randomization (MR) analyses showed an association between genetic predisposition to higher coffee consumption and increased SIS. Stratification analyses further explored differences in genetic associations across varying coffee consumption levels. Among individuals consuming coffee more than twice daily, two coffee consumption-associated single nucleotide polymorphisms (SNPs) in *AHR* and *CYP1A1*/*CYP1A2* were correlated with SIS. Integrative metabolomics and proteomics analyses identified lipid-related metabolites (triglycerides, phospholipids, free cholesterol) and inflammation-related proteins (DLK1, IL1RL2, CCL17) associated with the genetic proxy of coffee consumption. These findings suggest that genetically influenced coffee consumption may be associated with coronary atherosclerosis risk in frequent coffee drinkers, although the underlying biological basis remains to be clarified.

**Supplementary Information:**

The online version contains supplementary material available at 10.1038/s41598-026-44122-2.

## Introduction

Cardiovascular diseases (CVD) are a major global health concern^1^. Atherosclerosis, an inflammatory arterial disease, is the major cause of CVD^2^. Atherosclerosis in the coronary and carotid arteries is a common form of CVD, which might ultimately lead to the clinical complications including myocardial infarction (MI) and stroke. Advancements in imaging technology have enabled the noninvasive visualization of atherosclerotic plaques^3,4^. These advancements allow for a more accurate assessment of early CVD risk in cohort studies targeting populations without diagnosed disease. Coronary artery calcium score (CACS) derived from non-contrast computed tomography serves as a subclinical marker for atherosclerosis. In addition, since a CACS of 0 does not exclude atherosclerosis, the Segment Involvement Score (SIS), obtained by contrast-enhanced coronary computed tomography angiography (CCTA), has increased the precision of coronary atherosclerosis detection^5^. In predicting cardiovascular events, ultrasound-obtained carotid plaque measurements have also been proven valuable^6,7^.

Dietary factors are etiologically linked to the development of atherosclerosis and CVD^8–10^. Amidst these factors, the association between coffee consumption, recognized as the most widely consumed beverage worldwide after water, and cardiovascular health remains an ongoing debate^11–13^. Epidemiological studies have investigated the association between coffee consumption and cardiovascular health^13,14^, as well as traditional risk factors including type 2 diabetes (T2D)^15^, blood pressure^16,17^, and lipid profiles^16,18^. A study reported that consumption of coffee is positively associated with subclinical atherosclerosis^13^. In a study based on midlife women, occasional coffee intake was also found to be associated with more subclinical atherosclerosis^19^. Nevertheless, there is a limited number of studies investigating the relationship between coffee consumption and atherosclerosis.

Individuals may experience varying physiological responses to coffee consumption based on genetic differences. Genetic variations associated with coffee consumption have been identified through population-based genome-wide association studies (GWAS). The SNPs identified in GWAS highlighted genetic regions involved in caffeine metabolism, such as *CYP1A2* (15q24) and *AHR* (7p21)^20,21^. CYP1A2, responsible for more than 95% of caffeine clearance from plasma, plays a key role in caffeine metabolism. Its activity varies among individuals, contributing to the recognized differences in caffeine metabolism^20^. *AHR* plays a regulatory role in modulating the activity of CYP1A2 enzyme^21^. SNPs near *AHR* and *CYP1A2* were identified to be associated with higher plasma caffeine levels, slower metabolism, and lower coffee consumption, suggesting that individuals with these alleles may need less caffeine to achieve the desired stimulant effect^20,21^.

To date, studies examining the interplay of genes related to coffee and cardiovascular health are limited. Mendelian randomization (MR) offers an approach to strengthen causal inference by reducing confounding, thereby contributing to the assessment of potential associations. A case control study of Hispanic American descent (*n* = 4028) suggested that the risk of nonfatal MI depended on the ability to metabolize caffeine and that the carriers of variant CYP1A2*1F, the slow metabolizers, had an increased risk of MI with higher coffee consumption^22^. However, a more recent study (*n* = 347,077) found no evidence that genetic variants affecting caffeine metabolism modified the association between coffee consumption and cardiovascular diseases^23^. The findings across these two studies are not concordant. Regarding atherosclerosis, a study in 2024 demonstrated that genetically influenced coffee consumption increased the risk of CACS by two sample MR integrating GWAS results for CACS and coffee consumption from different studies^24^. However, more comprehensive and systematic assessments of subclinical atherosclerosis remain limited, with relatively limited use of omics-based analyses to provide insights into potential mechanisms, as well as a lack of individual-level paired data linking coffee consumption and atherosclerosis.

This study aims to analyze the association between coffee consumption and genetic predispositions influencing atherosclerosis in a Swedish middle-aged population. SIS, CACS and carotid plaque were evaluated as the outcomes to achieve a comprehensive assessment of atherosclerosis. To our knowledge, the association between coffee consumption and cardiovascular health, as reflected by these three outcomes, has not been extensively investigated. Given that Sweden ranks among the countries with the highest per capita coffee consumption worldwide^25^, our cohort exhibits a diverse range of coffee consumption patterns. Additionally, this study seeks to contribute to the limited body of research examining the interplay between genes related to coffee consumption and their influence on cardiovascular health. MR, as a method that can help strengthen causal inference under specific assumptions, was also employed. Furthermore, we aim to provide exploratory molecular context for these associations using proteomics and metabolomics analyses.

## Methods

### Data collection in SCAPIS

This study utilized data from the Swedish CArdioPulmonary bioImage Study (SCAPIS), a national large-scale observational study that randomly recruited 30,026 individuals aged 50–64 from the population in Sweden (https://www.scapis.org/). Participants were recruited from six major Swedish cities, including Uppsala, Umeå, Linköping, Göteborg, Malmö/Lund and Stockholm between 2013 and 2018^5,26^. The examinations were conducted at the six corresponding university hospitals, and all the examinations were performed on either two or three occasions within a two-week period. No exclusion criteria were applied when recruiting participants, except for the inability to understand spoken and written Swedish for informed consent^26^.

A comprehensive 140-question survey was designed to gather detailed data on self-reported health, family history, medication, occupational and environmental exposure, lifestyle, psychosocial well-being, socio-economic status, and other social determinants. Additionally, a 35-question food-frequency questionnaire (Mini-Meal-Q) was utilized^26^.

A 100 mL venous blood sample was collected from participants after an overnight fast for immediate analysis and biobank storage for future analysis^26^.

All SCAPIS participants provided written informed consent. Ethical approval for this study was obtained from the Swedish Ethical Review Board (2023–01559-01).

To minimize population stratification and ensure the validity and generalizability of the results, we focused on the analysis of participants of European ancestry. The selection was based on a principal component analysis (PCA) that combined the genome data from SCAPIS with the 1000 Genomes (1000G) (https://www.ebi.ac.uk/eva/?eva-study=PRJEB30460) and also accounted for participants’ parental birth countries. In total, 24,835 individuals with European ancestry were included in this study.

### Assessment of coffee consumption

A semiquantitative food frequency-questionnaire consisting of 35 questions, known as the Mini-Meal-Q, was employed to gather dietary information, including coffee consumption. Participants were inquired about the frequency at which they typically consume coffee, provided they drank it at least once a month. The available frequency options included never, 1–3 times a month, 1–2, 3–4, 5–6 times a week or 1,2,3,4,5 times a day. These options were converted into continuous numbers and estimated as average daily levels, as follows: 0, 0.067, 0.21, 0.50, 0.79, 1, 2, 3, 4 and 5 times per day, respectively.

### SIS measurements

SIS is a semiquantitative measure of the atherosclerosis burden in the coronary arteries, calculated using contrast-enhanced CCTA. In SCAPIS, cardiac imaging was assessed using electrocardiogram gated Computed tomography (CT) imaging at 120 kV. The CT that was obtained was a dual source CT scanner equipped with a stellar detector, (Somatom Definition Flash, Siemens Medical Solution, Forchheim, Germany). In preparation for imaging of SIS, renal function was elevated to identify contraindications and exclude participants at risk of contrast media. The participants also received beta blockers for heart rate deducing purpose, sublingual nitroglycerin for inducing vasodilation and an intravenous injection with contrast in preparation for CCTA^5^.

SIS has been demonstrated to be a strong and independent predictor of cardiovascular events and has shown superior efficacy in detecting atherosclerosis compared to CACS. The measurement reflects the total extent of coronary segments affected by atherosclerosis, regardless of the stenosis severity, with SIS representing the number of involved segments^5,27^.

### CACS measurements

CACS is a quantitative measure of calcium deposits in the coronary arteries, serving as an indicator of the burden and risk of coronary atherosclerosis. An increasing plaque burden correlates with higher cardiovascular risk, and CACS is valuable for guiding medical treatment and primary prevention strategies in high-risk asymptomatic patients^28^. Therefore, clinical significance of CACS has incremental prognostic value over conventional risk stratification when predicting future cardiovascular events^29^ and prediction of all-cause mortality^30^, making the measurement a strong and independent marker for subclinical heart disease^31^.

Images for analyzing CACS were obtained by using the same CT scan as for SIS, but without contrast media^5^. Based on the density and area of the calcium deposits on CT images, the deposits can be quantified using a scoring system, Agaston scoring system^32–34^.

### Carotid plaque measurements

Carotid plaque is a useful marker for cardiovascular risk since the measurement reflects not only the atherosclerotic process in the coronary arteries but also the atherosclerotic burden in other parts of the body. The noninvasive measurement of carotid plaque score is considered a strong predictor of major cardiovascular events^35^ and an independent risk factor for all causes and cardiovascular mortality^6^.

Atherosclerosis in the carotid arteries were imaged using the two-dimensional greyscale ultrasound (Acuson S2000 ultrasound scanner equipped with a 9L4 linear transducer) and ultrasound images were analyzed shortly after each examination by several operators. The assessment of plaque findings was categorized into three groups: no carotid plaque, unilateral carotid plaque/s and bilateral carotid plaques^26^. In the analysis, the carotid plaque measurements were converted into numerical values of 0, 1 and 2 respectively.

### Genome-wide SNP genotyping

Whole blood samples from different collection sites were randomized before DNA extraction to reduce extraction bias. Two robots (Hamilton Chemagic Star with Chemagen 96-head) performed DNA extraction via magnetic bead separation using Perkin Elmer reagent kit (CMG-1765-A, Lot No. B400-0126110). The quantity of extracted DNA was determined by UV spectrum analysis with Trinean DropSense 96 Polychromatic microplate reader (Techtum).

The DNA samples were genotyped using a customized version of the Illumina GSA-MDv3 at the SNP&SEQ Technology Platform in Uppsala. Genotypes were called with the GenomeStudio software (2.0.3). Finally, the imputed genotype dataset was prepared based on the Haplotype Reference Consortium r1.1 reference panel. The quality control (QC) for genetics data includes (i) genotyping calling rate > 98, (ii) unrelated samples to the 3^rd^ degree, (iii) adjusted heterozygosity falling within three standard deviations of the mean, (iv) no sex test errors and no sex chromosome aneuploidies.

In genomic data preprocessing, SNPs were kept based on an information score greater than 0.7, Hardy–Weinberg equilibrium *p* greater than 1e-6, and a minor allele frequency threshold of greater than 0.01 was applied to exclude rare variants. Additionally, SNPs and samples with a missing rate of less than 5% were included. In the end, a total of 6.9 million SNPs remained for subsequent analysis.

### Targeted proteomics

Proteomic data were generated from EDTA plasma samples with the Olink platform (Olink Proteomics, Uppsala, Sweden). A total of 184 proteins in panel Olink Target 96 Cardiovascular II (v. 5003) and Olink Target 96 Cardiovascular III (v. 6111) were measured using Proximity Extension Assay. The data was presented as normalized protein expression values. The inclusion criteria for proteomic analysis included obtaining completed informed consent and having performed: cardiac computed tomography angiography (CCTA) (or not performed CCTA due to low estimated glomerular filtration rate, estimated glomerular filtration rate (eGFR, < 50 ml/min)), computed tomography (CT) chest, ultrasound carotid arteries, accelerometer data and a complete questionnaire on smoking. The samples were included in a consecutive manner, beginning with the earliest available date for each site.

### Nuclear magnetic resonance-based metabolomics

The high-throughput nuclear magnetic resonance (NMR) metabolomics platform Nightingale (Helsinki-Finland, https://nightingalehealth.com)^36^ was applied to measure the plasma levels of 225 biomarkers including lipoprotein subclasses, lipids and small molecules in EDTA baseline plasma samples. More specifically, the Nightingale panel included (i) absolute quantitative measurements of lipids , lipoproteins, lipid content in lipoprotein particles, and small metabolites, all presented in mmol/L and (ii) the relative measurements of lipid content in each lipoprotein, defined as the lipid fraction relative to the total lipid content within the lipoprotein.

The inclusion criteria for metabolomic profiling were identical to those for proteomic analysis, ensuring a consistent approach to participant selection.

### GWAS of coffee consumption

PLINK2 (v 2.00-alpha-2–20190429) was applied to perform GWAS of coffee consumption in SCAPIS. The minimum frequency of SNP was set as 0.01. The covariates included sex, age, site, the first ten principal components of the genome, body mass index (BMI), smoking status, educational level, alcohol consumption habit, physical activity level and marital status. The same set of covariates was applied uniformly across all SNP-level association tests. For the GWAS results of males and females, the analysis was conducted separately for male and female participants, while keeping all other parameters consistent. The Manhattan plots were generated using R package CMplot (v 4.5.1).

The relationship between rs4410790/rs2472297 and coffee consumption was validated by general linear models, with sex, age, BMI, site, smoking status, educational level, alcohol consumption habit, physical activity level and marital status as covariates. The calculation and visualization of coffee consumption partial residuals were performed by R package visreg (v 2.7.0).

The linkage disequilibrium correlation information was extracted from the 1000 Genomes phase 3 reference panel (European population) by ieugwasr package (v 1.0.1).

The GWAS of coffee consumption in UK Biobank was sourced from previously published research results^37^. In this research, analysis was conducted on a homogeneous European ancestry population (*n* = 455,146). The covariates included genotyping array and the first 10 genetic principal components.

### Mendelian randomization analysis

MR analysis was conducted under three core instrumental variable assumptions: relevance (the instrument is associated with the exposure), independence (the instrument is independent of confounders), and exclusion restriction (the instrument affects the outcome through the exposure). For sensitivity analyses of one-sample MR, an endogeneity test was conducted under the assumption that the exposure is exogenous, and an overidentification test was performed under the assumption that all instruments are valid and consistent with the assumptions of independence and exclusion restriction. And for two-sample MR, sensitivity analyses were conducted under the assumptions that no single instrument unduly influences the estimate (leave-one-out), that instruments affect the outcome only through the exposure (horizontal pleiotropy analysis), and that effect estimates are consistent across instruments (heterogeneity analysis).

Carotid plaque was transformed into a continuous variable with the same method applied in the observational analysis. CACS was divided by 100 before one-sample Mendelian randomization (MR) to place the coefficients on a comparable scale for visualization. One-sample MR was performed by AER package (v 1.2–10, ivreg function), and rs4410790, as well as rs2472297 were instrumental variables (r^2^ < 0.01 and a 500 Kb window). Sex, age, site and the first ten principal components of genome were included as covariates to maintain the consistency of MR estimation. Two-stage least squares estimation was used as the main statistical model. The weak instrument test, endogeneity test, and overidentification test were provided through the diagnostic summary of the model constructed with the ivreg function. The power calculation for MR estimation was performed with an online tool (https://shiny.cnsgenomics.com/mRnd/)^38^. The phenotypes associated with the SNPs were retrieved from the GWAS Catalog (https://www.ebi.ac.uk/gwas/home).

For two-sample MR, the significant SNPs associated with coffee consumption in the UK biobank were clumped with a 1000 Kb window and a maximum r^2^ of 0.001 (the 1000 Genomes phase 3 reference panel [European population]). After clumping, a total of 30 SNPs remained that were shared between the two cohorts. The summarized level GWAS results between the 30 SNPs and three atherosclerosis indicators of SCAPIS were generated by PLINK2 (v 2.00-alpha-2–20190429), and the covariates included sex, age, site and the first ten principal components in the analysis. MRPRESSO (v 1.0) was applied to identify outliers before estimation. Before and after removing outliers, two-sample MR was performed with TwoSampleMR package (v 0.6.4). The inverse-variance weighted (IVW) method and MR-Egger were selected as the estimation model. The three sensitivity analyses, including leave one out sensitivity analysis, horizontal pleiotropy analysis and heterogeneity analysis, were conducted with TwoSampleMR package (v 0.6.4). The single SNP analysis and forest plot visualization were performed with TwoSampleMR package (v 0.6.4).

### Genetic risk score construction and stratification analysis

Among the significant SNPs that were associated with coffee consumption in SCAPIS, rs4410790 and rs2472297 were retained after clumping. The genetic risk score (GRS) for coffee consumption was calculated by summing the products of each participant’s effect allele counts at the rs4410790 and rs2472297 loci and the respective coefficients derived from the SCAPIS coffee GWAS. GRS was positively correlated with coffee consumption frequency (the Pearson correlation coefficient of 0.10, *p* value < 2.2e-16). The relationships between GRS and three atherosclerosis indicators were estimated using general linear models, separately at different coffee consumption levels, and sex, age, BMI, site, smoking status, educational level, alcohol consumption habit, physical activity level and marital status were adjusted in the estimation. The generalized additive model was applied to investigate the relationship between SIS and the interaction of coffee consumption and GRS, using a tensor product smooth with a basis dimension of 3. The same covariates were adjusted for in the generalized additive model. The partial residuals were calculated and visualized with visreg (v 2.7.0).

### Multi-omics network analysis

The relationships between GRS and measured proteins/metabolites were estimated using general linear models, with covariates adjusted consistently with those in stratification analysis. Proteins and metabolites with a *p* value of less than 0.025 were identified as associated with GRS, resulting in the selection of 6 proteins and 54 metabolites, and further network analysis was conducted on these proteins and metabolites.

The pQTL annotations for the 6 proteins were retrieved from https://metabolomics.helmholtz-munich.de/ukbbpgwas/^39^. The *p* value threshold was set as 5e-8. Linkage disequilibrium between the reported pQTLs and the variants used to construct the GRS was assessed using the 1000 Genomes phase 3 reference panel (European population) via ieugwasr package (v 1.0.1).

The associations between proteins and metabolites were identified with general linear models, and sex, age, as well as sites were adjusted for. The Benjamini–Hochberg method was employed to conduct multiple hypothesis testing, adjusting the *p* value to control the false discovery rate (FDR). Associations with FDR of less than 1e-6 were retained. Package igraph (v 2.0.2) was used to construct the network, calculate the degree of each protein/metabolite, and identify the communities (using the fast greedy algorithm and utilizing the absolute values of the regression coefficients as weights). The community structure provided a compact, descriptive summary to organize the protein-metabolite associations and to inform community-level annotation. Package qgraph (v 1.9.8) was applied to implement the Fruchterman-Reingold layout algorithm, and the visualization of network was completed with igraph. The association between proteins/metabolites and SIS was estimated using a general linear regression model, adjusting for the effects of age, sex, and site. Proteins with a *p* value of less than 0.05 and metabolites with a *p* value of less than 0.01 were selected for visualization in forest plots. This selection involved two steps: applying the *p* value criteria and ensuring that the associations with SIS and GRS were directionally consistent. In forest plots, the proteins and metabolites were ranked in descending order based on their degree in the network analysis. The calculation and visualization of SIS partial residuals were conducted with visreg (v 2.7.0).

### Statistical analysis

Participants with missing values in any of the measurements or covariates were excluded from the corresponding analysis. General linear models were applied to estimate the effect of coffee consumption on clinical indicators (stats package, R, v 4.3.1). The values of clinical indicators were standardized to z-scores (mean‑centered and scaled by the sample standard deviation) before analyzing. The models were adjusted for sex, age, BMI, site, smoking status, educational level, alcohol consumption habit, physical activity level and marital status. The forest plot was generated using the forestplot package (v 3.1.3) in R.

For the observational association analysis between coffee consumption and three atherosclerosis outcomes, to ensure that the estimated coefficients are on a comparable scale for visualization, CACS was divided by 100 before analysis for linear estimation. For carotid plaque, the absence of plaque was transformed to 0, while unilateral and bilateral carotid plaques were transformed to 1 and 2, respectively. For linear and non-linear estimation, general linear models (stats package, R, v 4.3.1) and generalized additive models (mgcv package, v 1.8–42) were applied. For the multivariable adjusted linear models and non-linear models, sex, age, BMI, site, smoking status, educational level, alcohol consumption habit, physical activity level and marital status were set as covariates. For non-linear models, partial residuals were calculated and visualized by visreg package (v 2.7.0).

## Results

### Coffee consumption and atherosclerosis indicators in SCAPIS European cohort

**Fig. 1:**
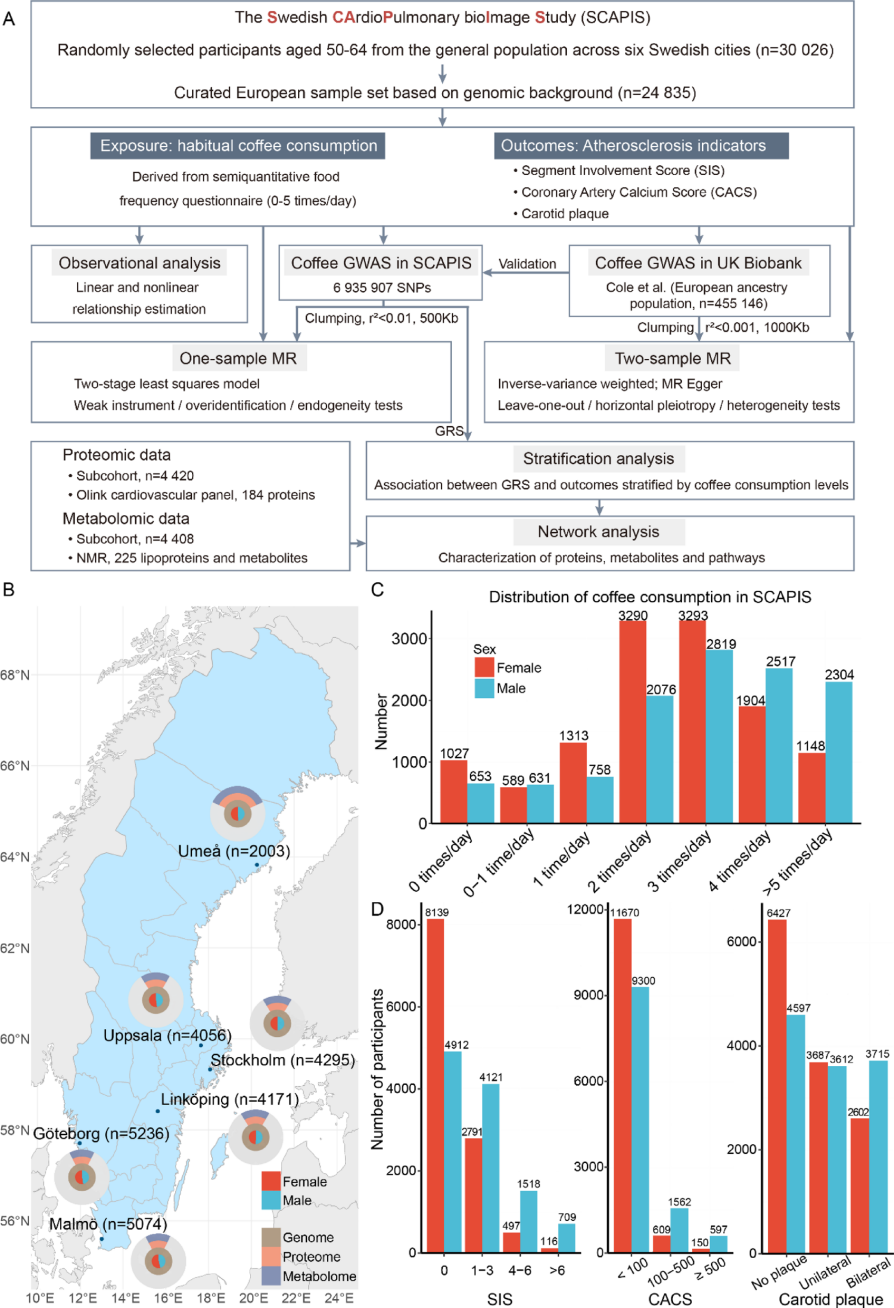
Coffee consumption and atherosclerosis indicators in SCAPIS. (**A**) Flowchart illustrating the study design and analysis flow. In this study, we performed observational analyses and MR to estimate the associations of coffee consumption and genetically influenced coffee consumption with three atherosclerosis outcomes, and stratification analysis was conducted to further investigate the interactive effect of genetic variations and coffee consumption. (**B**) Distribution of participants across six sites in Sweden, with pie charts displaying the available multi-omics data. (**C**) Overview of habitual coffee consumption amount in SCAPIS. (**D**) Distribution of SIS, CACS and carotid plaque in SCAPIS. CACS, Coronary artery calcium score; GRS, Genetic risk score; GWAS, Genome-wide association study; MR, Mendelian randomization; NMR, Nuclear magnetic resonance; SCAPIS, The Swedish CArdioPulmonary bioImage Study; SIS, Segment involvement score.

A total of 24,835 adults aged 50–64 from the SCAPIS European cohort was analyzed in the study (see more details in Methods) (Fig. 1A). The baseline information of participants, including sex, age, BMI, recruitment site, frequency of coffee consumption, smoking habit, alcohol consumption pattern, physical activity intensity, educational level and marital status, were collected (Table S1). Participants were recruited from six sites across Sweden, with an equal distribution of males and females at each site (Fig. 1B). The participants with proteomic and metabolomic profiles were also evenly distributed across different sexes and recruitment sites, supporting the representativeness of this study. The range of coffee consumption frequency spanned from zero to more than 5 times per day (Fig. 1C). The majority (*n* = 21,422, 88.1%) drank coffee at least one time a day and the most common frequency was 2–3 times each day, and males tended to drink higher amount of coffee than females. Observational analysis indicated that coffee intake was significantly correlated with several clinical variables, including lipid profiles (triglycerides, cholesterol, low-density lipoprotein and high-density lipoprotein), CVD biomarkers (N-terminal pro b-type natriuretic peptide [NTproBNP]), blood glucose, insulin, and systolic blood pressure (Figure S1A).

We further assessed the three subclinical atherosclerosis indicators (SIS [*n* = 22,803], CACS [*n* = 23,888] and carotid plaque [*n* = 24,640]) in SCAPIS (Fig. 1D). The percentage of individuals with SIS score of 1 or higher (indicating coronary stenosis in at least one coronary artery segment) was 42.8%. For CACS, 12.2% had a score of 100 or higher. Additionally, 55.3% had unilateral or bilateral carotid plaques. For all three outcomes, the atherosclerosis levels were higher in males compared to females, which were positively intercorrelated among the three indicators (Figure S1B). After adjusting for confounders, including sex, age, BMI, site, smoking status, education level, alcohol consumption habit, physical activity level and marital status, we estimated the effects of coffee consumption on the three atherosclerosis outcomes through both linear and nonlinear regression, none of the three atherosclerosis indicators were significantly associated with coffee consumption (Figure S1C, D). In the multivariable adjusted linear models (see more details in Methods), the coefficient estimates were -5.6e-3 (95% confidence interval [CI] -2.3e-2 to 1.2e-2) for SIS, -1.1e-2 (95% CI -3.1e-2 to 9.0e-3) for scaled CACS, and 5.6e-3 (95% CI -1.5e-3 to 1.3e-2) for carotid plaque.

### GWAS results of coffee consumption in SCAPIS

**Fig. 2:**
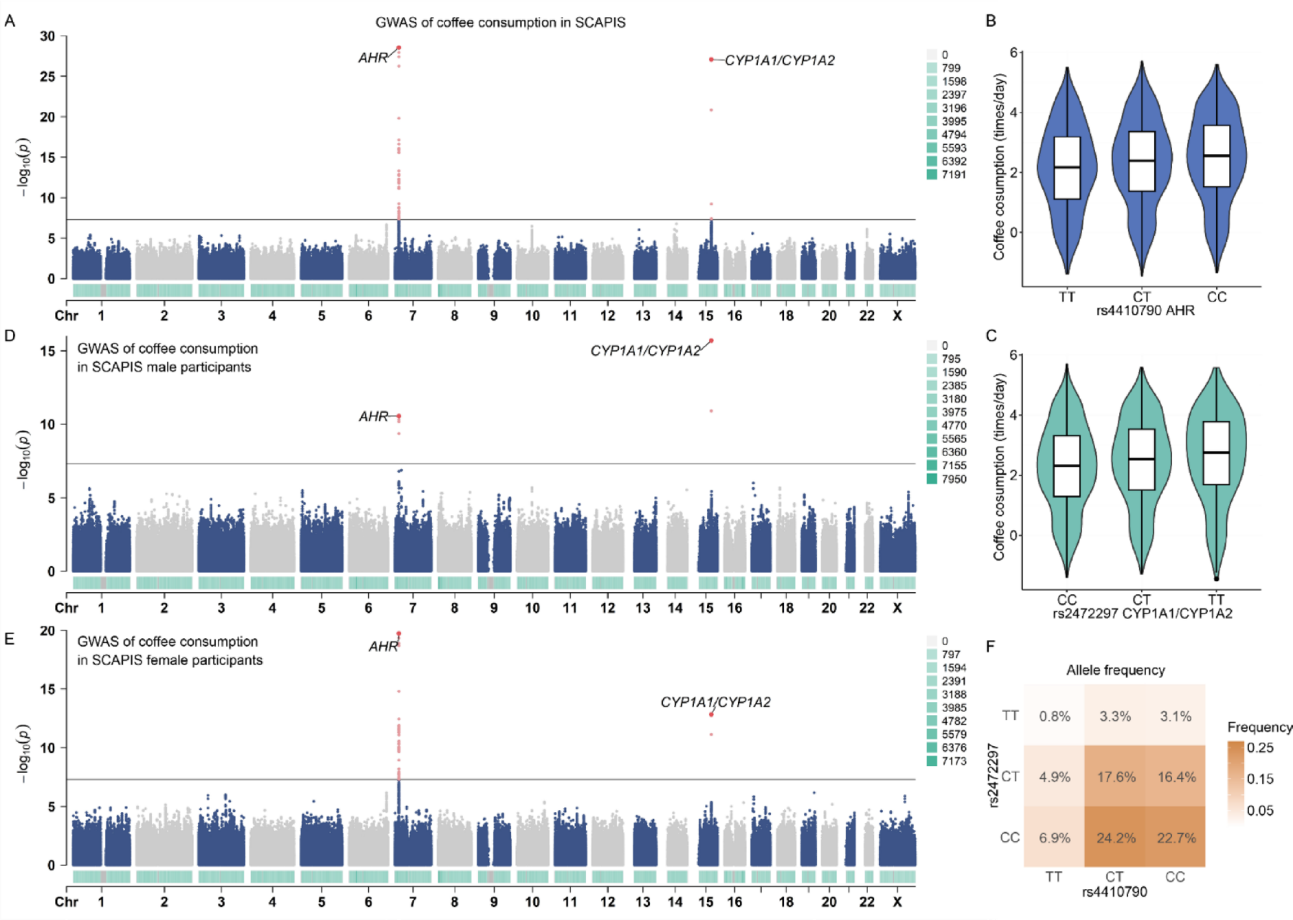
GWAS of habitual coffee consumption in SCAPIS. (**A**) Manhattan plot of coffee consumption in SCAPIS. The associations of rs4410790 (**B**) and rs2472297 (**C**) with coffee consumption amount were presented. Coffee consumption is displayed as partial residuals. (**D**) and (**E**) illustrate the Manhattan plots of coffee consumption in SCAPIS for males and females, respectively. (**F**) The combined allele frequency of rs4410790 and rs2472297 in SCAPIS. GWAS, Genome-wide association study; SCAPIS, The Swedish CArdioPulmonary bioImage Study.

To investigate the genetic factors associated with coffee consumption, we performed a GWAS of coffee consumption in SCAPIS (Fig. 2A, see more details in Methods). The two most significant associated SNPs were rs2472297 on chromosome 15q24 near *CYP1A2/CYP1A1* and rs4410790 on chromosome 7p21 near *AHR* (Fig. 2B, C). All significant SNPs (*p* value < 5e-8) on chromosome 7 exhibited an absolute linkage disequilibrium (LD) r-value greater than 0.15 with rs4410790, while those significant SNPs on chromosome 15 showed an absolute LD r-value greater than 0.3 (Figure S2A, B). Both rs4410790 and rs2472297 showed consistent associations with coffee consumption in males and females (Fig. 2D, E) and aligned with previous reports that these two variants were associated with habitual coffee consumption and caffeine metabolism rate in humans^20,37^. More than 95% of caffeine is metabolized by CYP1A2, and the aryl hydrocarbon receptor coded by *AHR* is also important in regulating CYP1A2 expression^20^. Additionally, rs2472297 resides in a region containing aryl hydrocarbon response elements by which AHR regulates CYP1A2 expression.

In SCAPIS, frequencies of C allele of rs4410790 and T allele of rs2472297, which were associated with higher coffee consumption, were 64.8% and 26.7%, respectively (Fig. 2F). Among individuals who drank coffee less than once per day, the frequencies of alleles associated with higher coffee consumption were lower (62.5% for rs4410790 and 25.3% for rs2472297), and for participants with coffee consumption exceeding 4 times per day, the corresponding allele frequencies were higher (69.2% for rs4410790 and 31.5% for rs2472297) (Figure S2C, D).

### Mendelian randomization estimation of genetically influenced coffee consumption and atherosclerosis outcomes

**Fig. 3:**
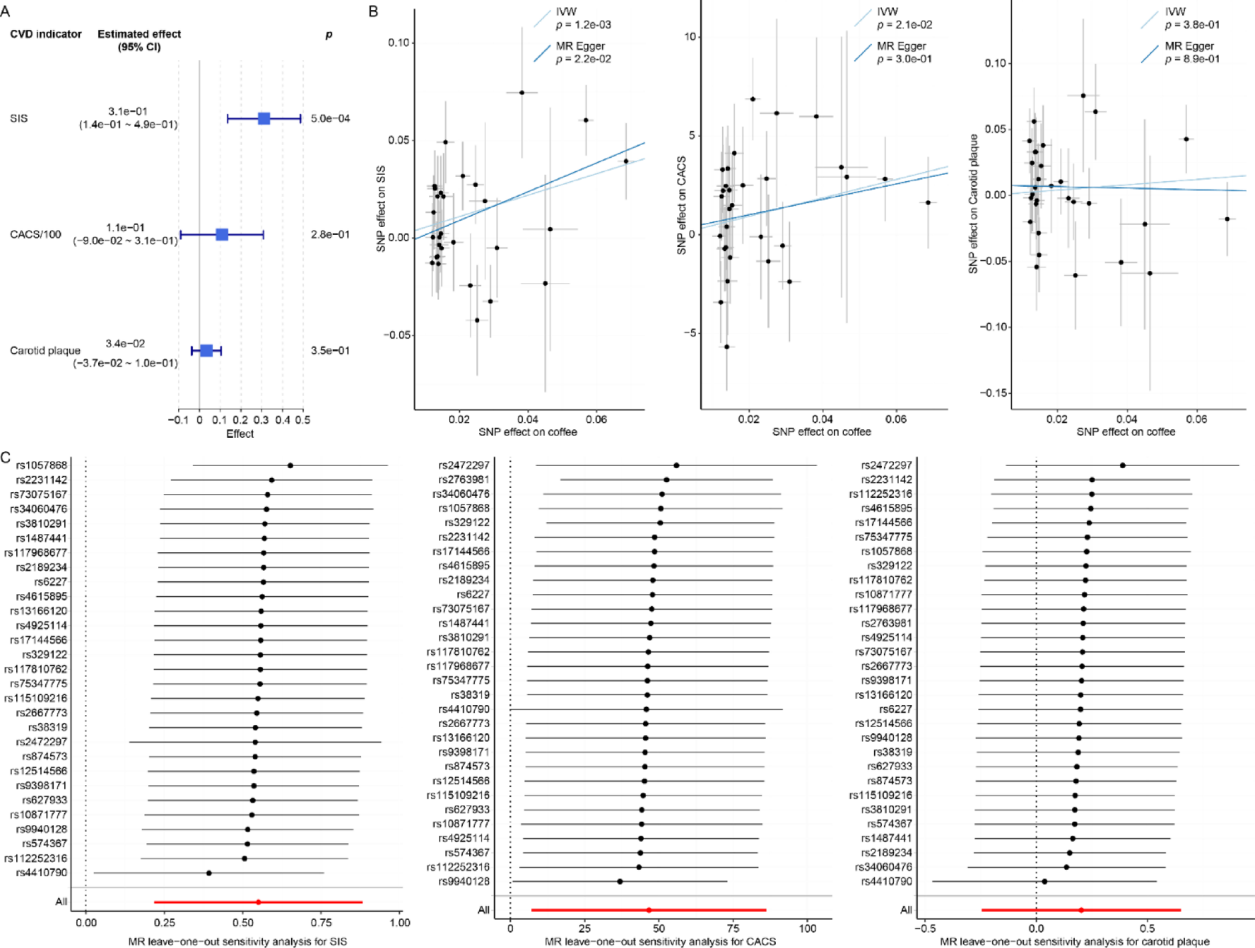
Mendelian randomization estimation between genetically influenced coffee intake and atherosclerosis outcomes. (**A**). One-sample MR estimation for relationship estimation between genetically influenced coffee intake and SIS, CACS as well as carotid plaque. The estimated effect represents the magnitude of change in the outcomes associated with each unit increase in genetically influenced coffee consumption (times/day). (**B**) Two-sample MR estimation with IVW and MR Egger models. Each point in the figures represents a single SNP. The horizontal and vertical axes indicate the effect sizes of the SNP on coffee consumption and the atherosclerosis outcomes, respectively. (**C**) MR leave-one-out sensitivity analysis for genetically influenced coffee consumption on outcomes. CACS, Coronary artery calcium score; CI, Confidence interval; CVD, Cardiovascular disease; IVW, Inverse-variance weighted; MR, Mendelian randomization; SIS, Segment involvement score; SNP, Single nucleotide polymorphism.

To assess whether genetic proxies for coffee consumption were associated with atherosclerosis, we employed one-sample MR analysis using two independent SNPs (rs4410790 and rs2472297, r^2^ < 0.01 and a 500 Kb window) as instrumental variables (Fig. 3A, see more details in Methods). These estimates reflect genetic predisposition to coffee consumption rather than the behavioral exposure itself. Among the three atherosclerosis indicators, SIS was positively correlated with genetically associated coffee consumption (the *p* value for SIS was 5.0e-4, the *p* values for CACS and carotid plaque were 0.28 and 0.35, respectively). Specifically, the estimated coefficient was 0.31 for SIS (95% confidence interval, 0.14, 0.49). For sensitivity analysis, the *p* value of weak instrument test was less than 2e-16 (F statistic 107), indicating adequate instrument strength. The *p* values of the endogeneity test and the overidentification test were 8.0e-04 and 0.21, respectively, indicating the appropriateness of the two-stage least squares estimator and the validity of the instruments, with no significant heterogeneity among the SNPs. The post hoc power calculations were as follows: *p* (SIS) = 0.95, *p* (CACS) = 0.20, *p* (carotid plaque) = 0.17.

Regarding the core assumptions of MR, the relevance assumption was supported by selecting SNPs identified and filtered through GWAS. Instrument strength was evaluated using weak instrument test (*p* < 2e-16, F = 107), indicating the selected variants were sufficiently associated with the coffee intake phenotype. For the independence assumption, the MR analysis incorporated covariates including sex, age, study site, and genomic principal components to account for potential confounding. Additionally, the selection of instrumental variables underwent rigorous clumping procedures to minimize linkage disequilibrium. Concerning the exclusion restriction assumption, the results of the overidentification test supported the consistency and validity of the selected instrumental variables. From a biological perspective, the chosen SNPs have been reported in previous studies to exhibit stronger associations with coffee intake-related phenotypes than with other traits, further substantiating their validity as instruments (Table S2). Additionally, we examined associations between the genetic instruments and questionnaire-derived dietary intake variables provided in the SCAPIS dataset, as well as cardiometabolic risk factors (e.g., BMI, SBP, and DBP). No clear evidence of strong associations was observed (Table S3). However, the exclusion restriction assumption cannot be definitively verified, particularly given the limited number of instrumental variables. Although no strong evidence of horizontal pleiotropy was observed, subtle pleiotropic effects cannot be entirely excluded. Therefore, further studies incorporating additional genetic instruments and more comprehensive investigations of the underlying biological mechanisms are needed for confirmation.

We next performed a two-sample MR using results from UK Biobank to validate the associations between genetically influenced coffee consumption and atherosclerosis indicators (Fig. 3B, S3). SCAPIS and UK Biobank participants were recruited independently in Sweden and the UK, respectively, with full anonymization procedures applied. There is no known sample overlap between the two cohorts. GWAS results from the UK Biobank for coffee intake identified rs4410790 and rs2472297 as the most strongly associated SNPs, which is consistent with findings from the SCAPIS cohort. Thirty independent SNPs were identified as instruments from GWAS results of coffee consumption in UK Biobank, and the associations between these SNPs and atherosclerosis indicators were identified in SCAPIS (Table S4). One SNP (rs2763981, *p* value for outlier test < 0.01) was identified as an outlier for SIS and no outliers were identified for CACS and carotid plaque. After removal of outliers, IVW model indicated that genetically influenced coffee consumption was positively correlated with SIS (estimate = 0.55, se = 0.17, *p* = 1.2e-3) and CACS (estimate = 46.66, se = 20.19, *p* = 2.1e-2). The result was supported by MR Egger model for SIS (estimate = 0.74, se = 0.31, *p* = 2.2e-2).

Meanwhile, before removing the single outlier for SIS, the IVW model suggested that genetically influenced coffee consumption was positively associated with SIS (estimate = 0.48, se = 0.20, *p* = 1.8e-2), and the MR Egger model yielded (estimate = 0.85, se = 0.36, *p* = 2.5e-2). These estimates were consistent with the main results obtained after outlier removal.

Three sensitivity analyses were then applied. The leave-one-out analysis can assess robustness by excluding each genetic instrument and re-estimating the effect (Fig. 3C). For SIS, the estimated effect was still significant after excluding any SNP, however for CACS, after excluding rs4410790 the effect was not significant. Therefore, the result of CACS was driven by specific variant. The second sensitivity analysis was for horizontal pleiotropy. The *p* values for SIS and CACS were 0.46 and 0.81. And for the third sensitivity analysis, heterogeneity test, the *p* values for SIS and CACS were 0.20 and 0.18, suggesting no strong evidence of pleiotropy or heterogeneity in either analysis. Therefore, sensitivity analysis supported the relationship between genetically influenced coffee consumption and SIS, while the result of CACS was biased.

Accordingly, both one-sample MR and two-sample MR demonstrated consistent results, suggesting a potential relationship between genetically influenced coffee consumption and SIS.

### Association between GRS and atherosclerosis outcomes stratified by coffee consumption levels

**Fig. 4:**
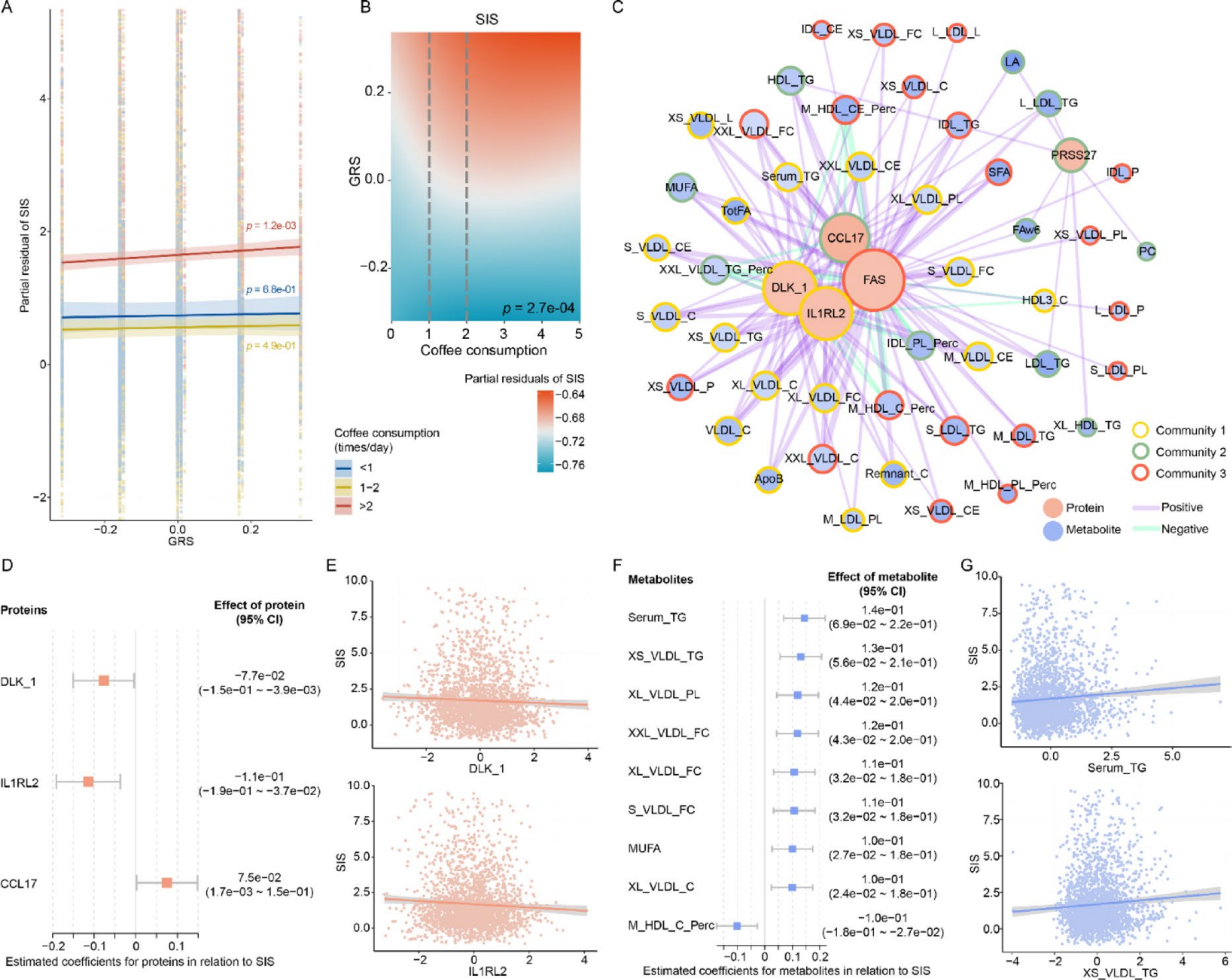
Association between GRS and SIS stratified by coffee consumption levels. (**A**) The association between GRS and SIS among participants with varying levels of coffee intake. For participants who drank coffee more than twice a day, coffee GRS was positively correlated with SIS. SIS is presented as partial residuals, with the visualization restricted to the range of  −2 to 5. (**B**) Nonlinear interaction analysis results of GRS and coffee consumption on SIS. (**C**) Network analysis of proteins and metabolites associated with GRS in participants who consumed coffee more than twice per day. The sizes of nodes represent the degree of proteins/metabolites within the network, indicating their connectivity in the overall interaction landscape. The increased thickness of the links in the network indicates a stronger correlation between proteins and metabolites. (**D**) The proteins which were related to SIS among the proteins in network. (**E**) The relationship between DLK1, IL1RL2 and SIS (SIS is presented as partial residuals). (**F**) The metabolites which were related to SIS among the metabolites in network. (**G**) The relationship between serum TG, XS_VLDL_TG and SIS (SIS is presented as partial residuals). CI, Confidence interval; GRS, Genetic risk score; SIS, Segment involvement score.

To further analyze the combined effects of coffee intake and genetic factors on atherosclerosis, we stratified participants in SCAPIS based on the frequencies of coffee consumption and examined the relationship between SIS and the GRS of coffee intake which was constructed based on rs4410790 and rs2472297 (see more details in Methods). For individuals consuming coffee no more than twice per day, GRS was not associated with SIS (Fig. 4A). While among those with a daily intake exceeding two times, a positive correlation was observed between GRS and SIS. A nonlinear model incorporating interaction terms of GRS and coffee intake yielded consistent results (Fig. 4B). No significant results were identified for CACS or carotid plaque (Figure S4A, B). The upper threshold was pre‑specified at > 2 times/day (i.e., ≥ 3 times/day) to approximate the upper tertile boundary (3 times/day) of coffee consumption, and to maintain adequate sample sizes across groups. In the full‑sample nonlinear model, the association pattern appeared comparatively stable beyond this level, suggesting a boundary for summarization (Fig. 4B). Further sensitivity analyses using alternative thresholds yielded consistent patterns (Table S5), indicating that the > 2 (≥ 3) threshold provides an appropriate summary of the observed stratum‑specific GRS–SIS association pattern.

We further conducted exploratory, hypothesis‑generating proteomics and metabolomics analyses to characterize molecular patterns associated with GRS among frequent coffee drinkers (more than two times per day). For participants who drank more than two times of coffee per day, 6 proteins and 54 metabolites associated with GRS were identified. No GRS component variant was a reported pQTL for the 6 proteins, nor was any in LD (r^2^ > 0.01) with the reported pQTLs (Table S6). A network analysis was further performed to explore proteins and metabolites associated with the GRS (Fig. 4C, Table S7 and S8, see more details in Methods). Using a fast greedy network analysis algorithm, we detected three densely connected communities within the protein-metabolite network. This community-level representation provided a compact summary of correlated structure and reflected the underlying organization of proteins and metabolites. Community 1 included Delta like non-canonical Notch ligand 1 (DLK1), interleukin 1 receptor like 2 (IL1RL2) and lipid/lipoprotein profiles such as Apolipoprotein B (ApoB), serum triglyceride (TG), remnant cholesterol, total fatty acids, as well as very low density lipoprotein (VLDL) components. Community 2 included C–C motif chemokine 17 (CCL17), serine protease 27 (PRSS27) and certain types of fatty acids (linoleic acid, LA; omega-6 fatty acid, FAw6 and monounsaturated fatty acid, MUFA), and community 3 included tumor necrosis factor receptor superfamily member 6 (FAS) and saturated fatty acids.

We then assessed the associations between these GRS-related proteins and metabolites and SIS (Fig. 4D-G). DLK1 and IL1RL2 in community 1 were negatively associated with both GRS and SIS, while CCL17 from community 2 showed a positive association with both GRS and SIS. The majority of metabolites that were associated with both GRS and SIS were located within community 1, consisting of serum TG and lipid components of VLDL. The TG in serum and free cholesterol, phospholipids, as well as triglycerides in different sizes of VLDL particles were positively associated with GRS, and these lipid indicators were also positively correlated with SIS.

DLK1 is a marker of precursor adipocytes, and is associated with fat metabolism and obesity^40,41^. Meanwhile, circulating levels of TG and VLDL components showed consistent associations with increasing GRS. Higher levels of TG was associated with subclinical atherosclerosis^42^. On the other hand, IL1RL2, also known as the IL-36 receptor, plays an important role in immune regulation. Additionally, CCL17, a pro-inflammatory mediator, has been found to have elevated expression in various inflammatory diseases, including atherosclerosis^43,44^. Therefore, among participants drinking coffee more than twice per day, we observed two network-defined molecular communities, one characterized by lipid-related molecules (community 1) and another characterized by inflammation-related proteins (community 2), in analyses involving both the GRS and SIS.

## Discussion

In this population-based cohort study, coffee consumption was not associated with three atherosclerosis outcomes in the observational analysis. However, MR estimation suggested a potential positive association between genetically influenced habitual coffee consumption and SIS. In participants who drank coffee more than twice daily, higher coffee GRS was associated with higher SIS, with lipid metabolism and inflammation related molecular features, including DLK1, IL1RL2 and CCL17, observed alongside this association.

The association between cardiovascular health and consumption of coffee, one of the most popular beverages worldwide, has been a persistent research topic of interest. Different components in coffee exhibited inconsistent effects on cardiovascular homeostasis, and previous reports showed complex regulatory roles of coffee intake in the context of cardiovascular health^45,46^. Higher coffee consumption was reported to be correlated with higher low density lipoprotein cholesterol (LDL-C) levels, high density lipoprotein cholesterol (HDL-C) levels, total cholesterol (TC) levels, ApoB as well as lower TG, systolic and diastolic blood pressure, and NTproBNP levels^14,16,18,47–49^. These findings on clinical profiles were largely consistent with this study, which confirmed the multiple regulatory effects of coffee consumption. The observational studies on the effects of coffee on CVD yielded heterogeneous results across different studies. The reported associations were J-shaped, positive or non-significant^16,23,50,51^. No significant association between coffee consumption and SIS, CACS and carotid plaque was found in the SCAPIS observational results.

The GWAS results identified and verified coffee consumption-associated SNPs from 2 genome regions, 7p21 and 15q24. The independent top SNPs from the two regions were rs4410790 and rs2472297, mapped to *AHR* and intergenic regions near *CYP1A1/CYP1A2*. CYP1A2 is an essential enzyme for caffeine metabolism and AHR is a regulator of CYP1A2. Meanwhile, rs2472297 and rs4410790 were also reported to be associated with caffeine metabolism rate in human bodies^20^. Caffeine metabolism is a potential key pathway through which genetic variants influence coffee consumption.

Two MR estimation methods suggested a potential positive correlation between genetically influenced coffee consumption and SIS, with sensitivity analyses yielding broadly consistent results. While the one-sample MR estimation might be susceptible to bias due to sample overlap, the results were broadly consistent with those obtained from a two-sample MR analysis conducted using an independent dataset. Of the previous MR analyses on coffee consumption and CVD, most studies reported a non-significant estimate^52–56^. A study in a European population background on coronary artery disease, which is highly associated with SIS, showed consistent results^57^.

MR estimation in this study is interpreted within the standard instrumental-variable framework and depends on three core assumptions: relevance (the genetic variants are associated with coffee consumption), independence (the variants are not related to major confounders of the coffee–atherosclerosis relationship), and the exclusion restriction (the variants influence atherosclerosis only through coffee consumption). The relevance assumption is supported by prior genome-wide association studies and by the observed instrument strength in our analyses, while the independence assumption is considered plausible given random allocation of genetic variants at conception, although residual associations with unmeasured confounders cannot be completely excluded.

The exclusion restriction assumption, however, warrants particular caution. While these assumptions are considered throughout the study and are evaluated where feasible, the exclusion restriction in particular cannot be established with complete certainty in applied settings, as the genetic variants may be associated with the outcome through alternative biological pathways, although the evaluations performed did not provide evidence for this. We therefore interpret the MR results cautiously and view them as supportive genetic evidence. They merit further evaluation in additional datasets using complementary approaches.

The divergence between the observational analyses and MR estimation warrants cautious interpretation. First, observational models may remain affected by residual confounding and exposure misclassification despite adjustment, particularly for behaviors such as coffee intake that correlate with multiple lifestyle and socioeconomic factors. Such bias can attenuate an association towards the null and may therefore partly explain the absence of a clear observational signal. Second, the MR instruments proxy a genetically influenced, lifelong component of coffee consumption, which may not align with the short-term or self-reported exposure measured in the observational setting; as a result, the MR estimate can reflect a different exposure construct and timing than the observational estimate. Third, limited power, driven both by the reliance on limited independent variants and by the available cohort sample size, reduces sensitivity to detect modest effects, and may contribute to variability in point estimates across approaches. Despite these limitations, the MR results may still be informative by offering an estimate that is generally less prone to classical confounding than conventional regression analyses. As such, they help motivate a cautious hypothesis that should be examined through triangulation with complementary approaches.

Furthermore, stratification analyses suggested a positive association between SIS and GRS among participants who consumed coffee more than twice per day (estimate of 0.37, 95% confidence interval, 0.14, 0.59), rather than those with lower coffee intake. Network analysis of proteomic and metabolomic profiles revealed molecular features observed alongside the positive relationship among participants with coffee consumption of more than twice a day. In this subgroup, higher coffee GRS was associated with higher levels of ApoB, serum TG, remnant cholesterol and total fatty acid levels. Moreover, DLK1, IL1RL2 and CCL17 were associated with both GRS and SIS. DLK1 functions as a growth regulator of cells including adipocytes and has potential cardio-protection effects^58^. IL1RL2 and CCL17 have been implicated in cardiovascular disease through their roles in inflammation and immune response regulation^44,59,60^. Specifically, CCL17 has been previously reported to be involved in the immune microenvironment in vascular dysfunction^60^. Accordingly, lipid metabolism and inflammation related molecular features showed associations with both the GRS and coronary artery atherosclerosis.

Several limitations should be acknowledged. First, the one-sample MR and GRS analyses relied on two independent SNPs. Accordingly, the GRS is not polygenic. This may reduce statistical power and increase sensitivity to instrument-specific properties. Notwithstanding these constraints, the selected variants were derived from GWAS-identified loci and align with the top coffee-related signals in the UK Biobank, and complementary two-sample MR analyses using additional UK Biobank instruments yielded estimates consistent with the one-sample results, supporting robustness and suggesting potential generalizability. Nevertheless, given the limited number of variants, the findings would benefit from further evaluation in additional datasets.

Second, although the coffee consumption phenotype showed no significant linear or nonlinear association with SIS in the observational analyses, stratifying by coffee consumption may still introduce potential collider bias, as coffee intake may be affected by both genetic liability and factors related to SIS. Conditioning on such a variable may induce associations within strata. Accordingly, the stratified findings are treated as complements to the full‑sample analyses. Meanwhile, the nonlinear interaction model showed consistent patterns, providing support for the observed association structure.

Third, because coffee consumption was self-reported, and we lacked information on cup size, coffee type (including decaffeinated), brewing method and caffeine content, future studies with more detailed questionnaires and objective measurements will help clarify the relationship between the compounds of coffee and atherosclerosis. As the findings were based on European populations, replication in other populations might be necessary to assess the broader applicability of the results.

## Conclusions

In conclusion, our findings suggest a potential positive association between genetically influenced coffee consumption and SIS among participants who consumed coffee more than twice a day. Multi-omics analyses revealed lipid metabolism and inflammation related molecular features observed alongside this association.

## Supplementary Information

Below is the link to the electronic supplementary material.

Supplementary Material 1

## Data Availability

The SCAPIS data used in this study were accessed under ethical approval from the Swedish Ethical Review Board and with permission from the SCAPIS Data Access Board. Due to the presence of sensitive personal information, the data are not publicly available. The pseudonymized SCAPIS phenotype and genotype data can be accessed by qualified researchers upon request. Access requires obtaining approval from the Swedish Ethical Review Board as well as approval from the SCAPIS Data Access Board (https://www.scapis.org/data-access). The GWAS summary statistics from the UK Biobank used in this study are publicly available at: http://www.kp4cd.org/dataset_downloads/t2d^37^.

## Abbreviations

ApoA1: Apolipoprotein A1
ApoB: Apolipoprotein B
BMI: Body mass index
CACS: Coronary artery calcium score
CCTA: Cardiac computed tomography angiography
CI: Confidence interval
CT: Computed tomography
CVD: Cardiovascular disease
DBP: Diastolic blood pressure
eGFR: Estimated glomerular filtration rate
FAw6: Omega-6 fatty acid
FDR: False discovery rate
GRS: Genetic risk score
GWAS: Genome-wide association study
HbA1c: Hemoglobin A1c
HDL: High density lipoprotein
HDL-C: High density lipoprotein cholesterol
IVW: Inverse-variance weighted
LA: Linoleic acid
LD: Linkage disequilibrium
LDL: Low density lipoprotein
LDL-C: Low density lipoprotein cholesterol
Lpa: Lipoprotein(a)
MI: Myocardial infarction
MR: Mendelian randomization
MUFA: Monounsaturated fatty acid
NMR: Nuclear magnetic resonance
NTproBNP: N-terminal pro b-type natriuretic peptide
PCA: Principal component analysis
QC: Quality control
SBP: Systolic blood pressure
SCAPIS: The Swedish CArdioPulmonary bioImage Study
SIS: Segment involvement score
SNP: Single nucleotide polymorphism
T2D: Type 2 diabetes
TC: Total cholesterol
TG: Triglyceride
VLDL: Very low density lipoprotein
