# Supplementary material for: Genetic predisposition to coffee consumption and the association with the early risk of atherosclerosis
Source: Sci Rep. 2026 Mar 22;16:9652. doi: 10.1038/s41598-026-44122-2 (PMC13009213; doi:10.1038/s41598-026-44122-2)

**Genetic predisposition to coffee consumption and the association with the early risk of atherosclerosis**

**Supplementary method**

**Covariate assessment**

The covariates included in this study were self-reported through questionnaires or obtained through registered information, physical examinations and monitoring.

The ages of participants at study visit were collected from registered information and rounded to 1 decimal. The sites of examination (Göteborg, Linköping, Malmö, Stockholm, Umeå and Uppsala) and the gender (man/woman) were also derived from registered information.

The self-reported questionnaire provided information on various characteristics, including the following: smoking status categorized as never smoker, former smoker and current smoker; frequency of having an alcoholic drink categorized as once a month or less, twice a month to three times a week, and four times a week or more; highest completed level of education categorized as no formal education, primary education, secondary education and higher education; marital status categorized as single, divorced, married and widowed.

Additionally, the physical examination included weight and height measurements, conducted according to current recommendations. The body mass index (BMI) was calculated by dividing the weight (kg) by the square of height (m^2^), expressed as kg/m^2^. The total minutes of moderate- and vigorous-intensity physical activity during waking hours over a 7-day period were recorded using tri-axial accelerometry (Actigraph GT3X and GT3X+).

**Clinical blood analysis and vital signs**

N-terminal pro b-type natriuretic peptide (NTproBNP) and Troponin I were measured in frozen EDTA plasma samples with Alere NT-proBNP for Alinity I, and Alinity I STAT hs Troponin I, respectively. The instrument for measurement was Alinity I, Abbott.

Lipid indicators including triglyceride (TG), cholesterol, low-density lipoprotein (LDL), high-density lipoprotein (HDL), were performed at each site with fresh blood samples after an over-night fast.

Apolipoprotein A1 (ApoA1), apolipoprotein B (ApoB) and lipoprotein(a) (Lpa) were analyzed from frozen EDTA plasma samples with Alinity c Apolipoprotein A1, Alinity c Apolipoprotein B, and Alinity c Lp(a) reagent using Alinity C, Abbott.

Pulse rate was recorded during the electrocardiogram measurement. Participants rested supine for five minutes to stabilize heart rate before recording. Brachial arterial blood pressure (systolic and diastolic blood pressure) was measured in both arms by OMRON M10-IT (Omron Healthcare Co. Ltd, Japan) after a 5-minute rest in a supine position. Measurements were repeated until two consecutive readings were within ±10 mmHg, with a maximum of four attempts. Systolic blood pressure (SBP) was calculated as the average of two measurements from the arm with the highest mean SBP. Diastolic blood pressure (DBP) was similarly averaged from the same arm, or from the right arm if mean SBPs were equal in both arms.

The capillary glucose level, as well as hemoglobin A1c (HbA1c) level were measured at each site with fresh blood samples after an over-night fast. Insulin was analysed in frozen EDTA plasma samples with Alinity I Insulin reagent by Alinity I, Abbott.


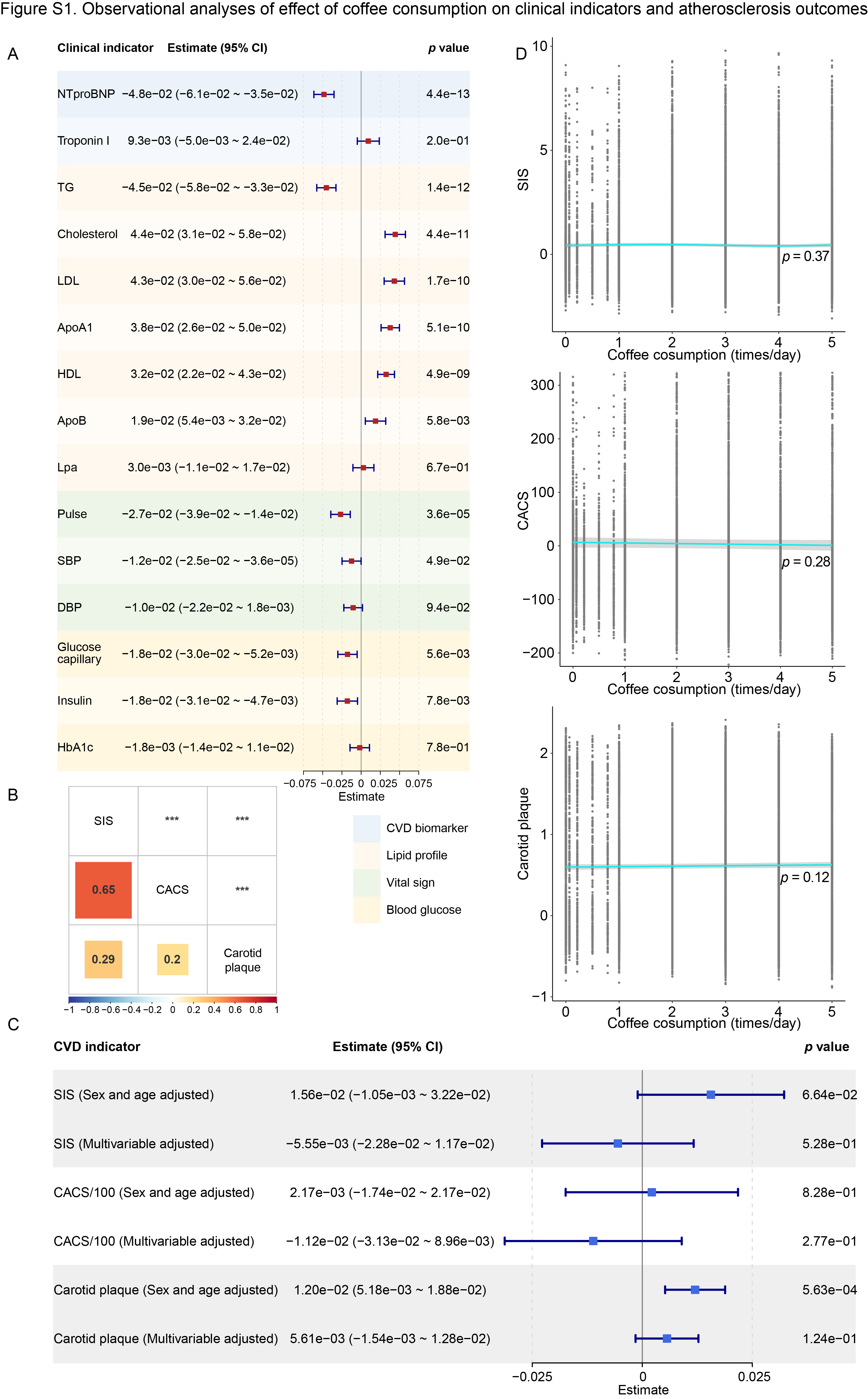


**Figure S1. Observational analyses of effect of coffee consumption on clinical indicators and atherosclerosis outcomes**

(A) The relationship between coffee consumption and clinical indicators. The estimated effect quantifies the increase in the clinical indicators in terms of standard deviations for each one-unit increase in coffee consumption (times/day). (B) The correlation matrix of atherosclerosis outcomes. (C) The effect of coffee consumption on outcomes, which was estimated with linear regression models. (D) The estimation of nonlinear effects of coffee consumption on the outcomes. The atherosclerosis outcomes are presented as partial residuals.


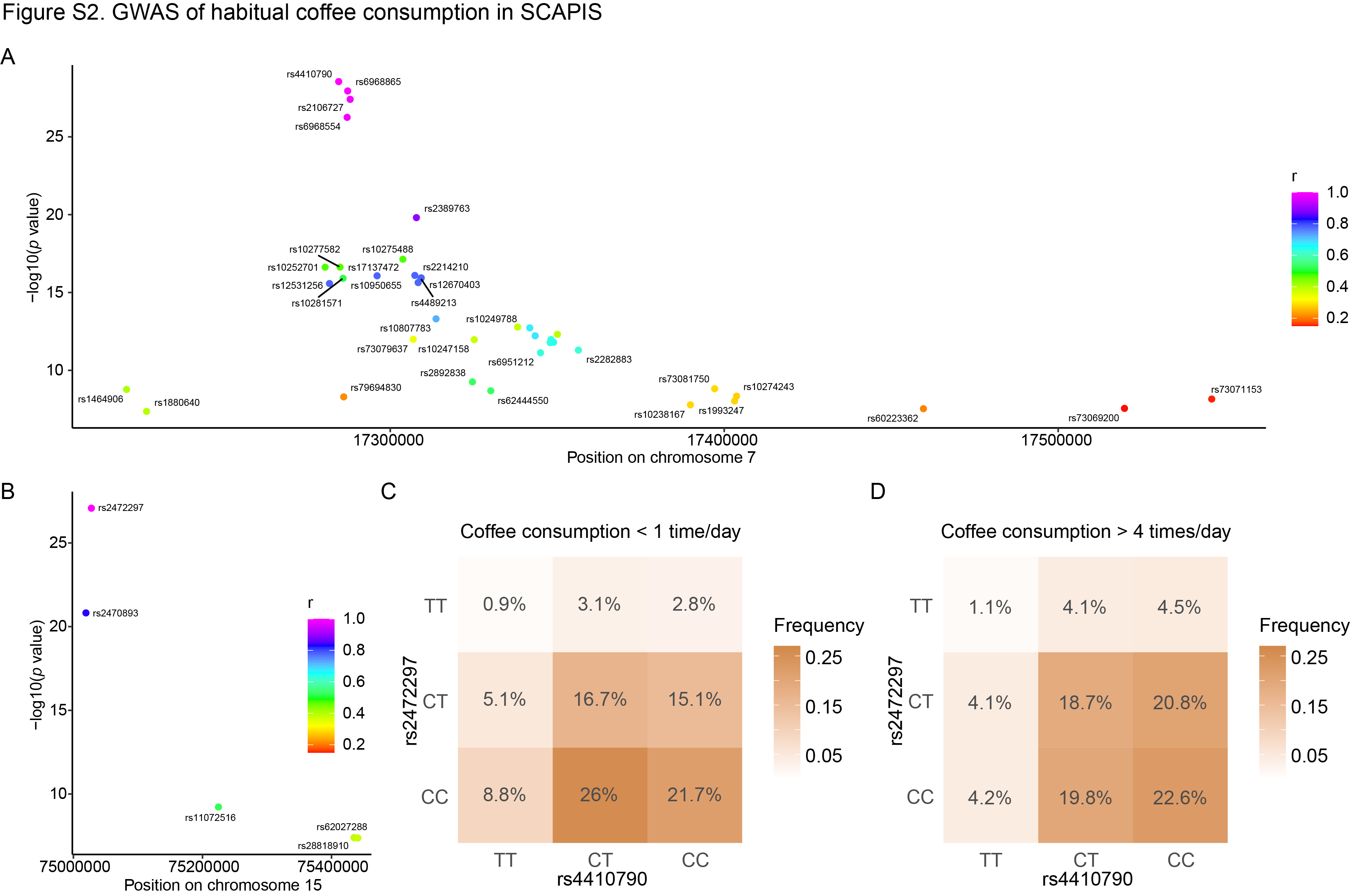


**Figure S2. GWAS of habitual coffee consumption in SCAPIS**

The linkage disequilibrium correlation between the significant SNPs on chromosome 7 and rs4410790 (A), as well as between the significant SNPs on chromosome 15 and rs2472297 (B). (C) and (D) depict the combined allele frequencies of rs4410790 and rs2472297 in SCAPIS, among participants who consume coffee less than once per day and those who consume it more than four times per day.


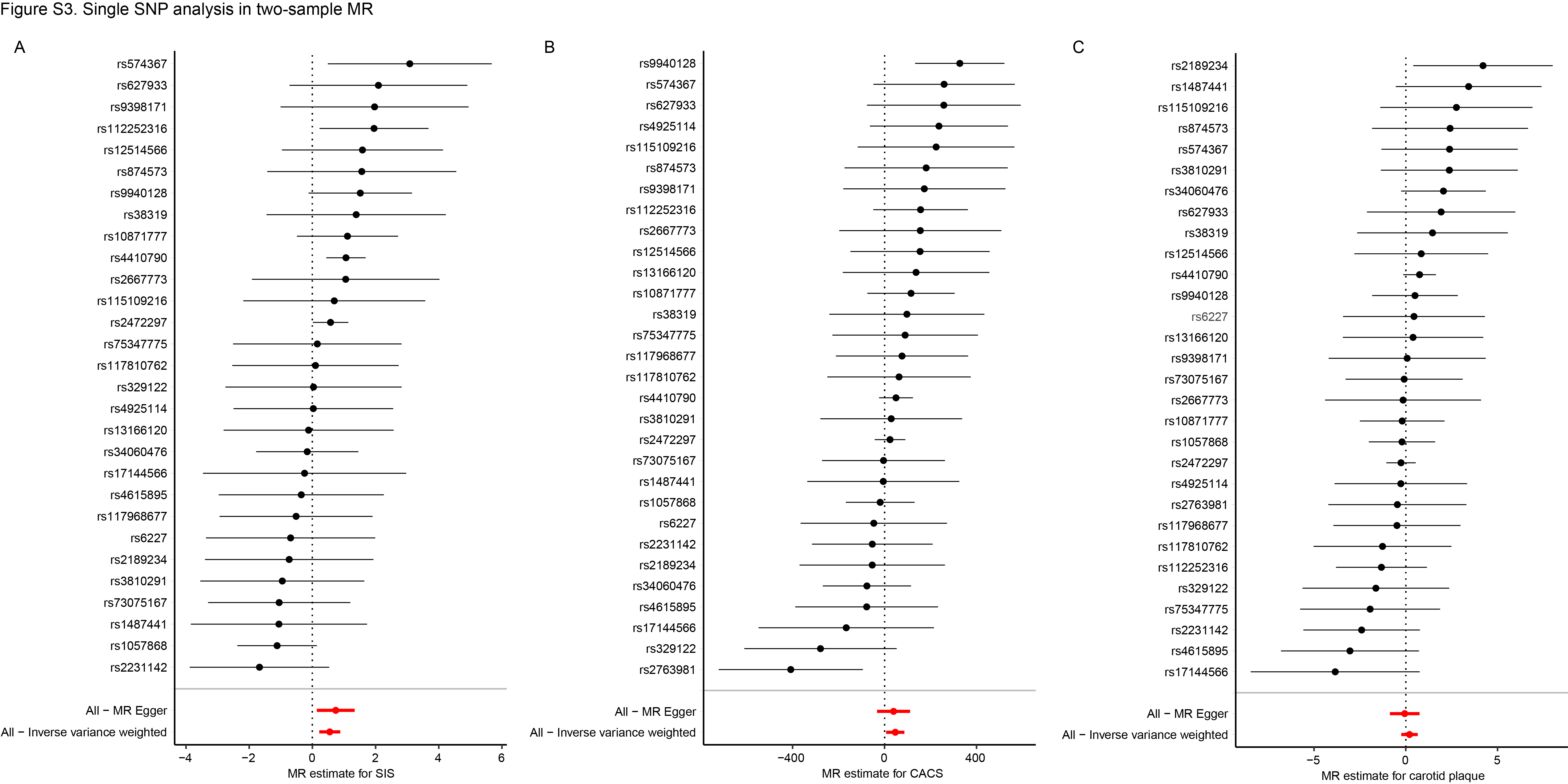


**Figure S3. Single SNP analysis in two-sample MR**

(A), (B) and (C) present the results of the relationship estimates from single SNP analyses of genetically influenced coffee consumption with SIS, CACS, and carotid plaque, respectively, in the two-sample MR analysis.


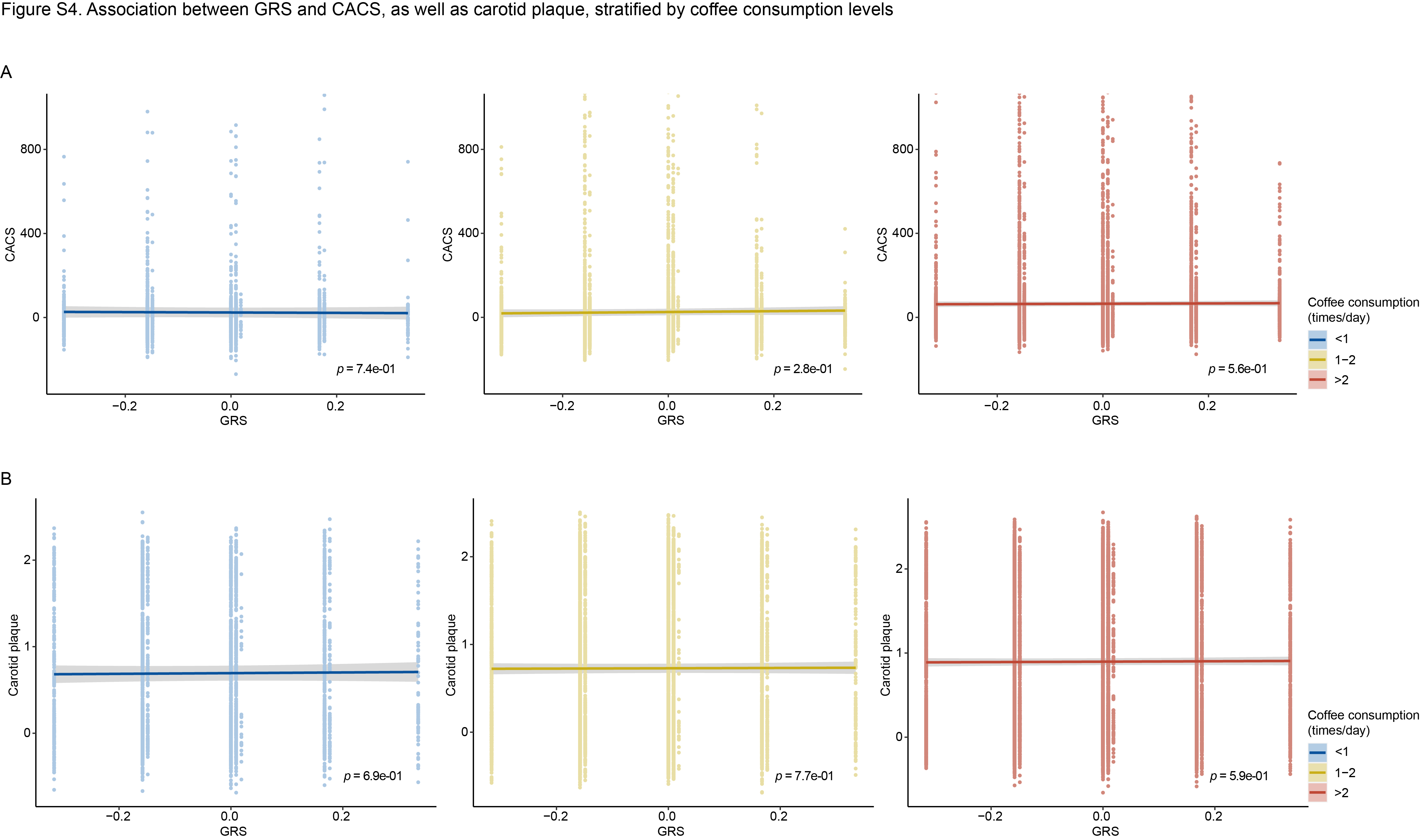


**Figure S4. Association between GRS and CACS, as well as carotid plaque, stratified by coffee consumption levels**

The association between GRS and CACS (A), as well as carotid plaque (B), among participants with varying levels of coffee intake. CACS and carotid plaque are presented as partial residuals.


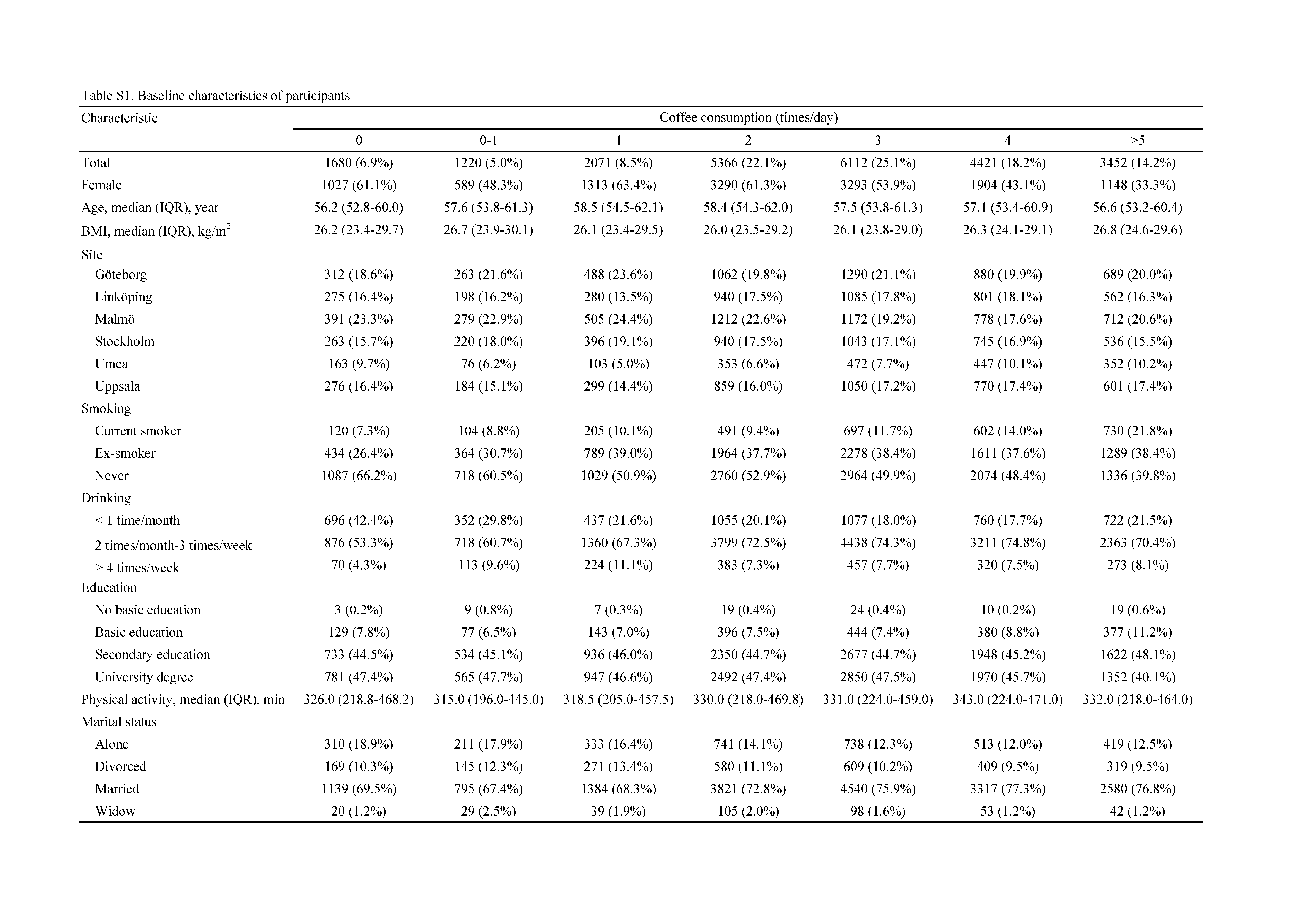


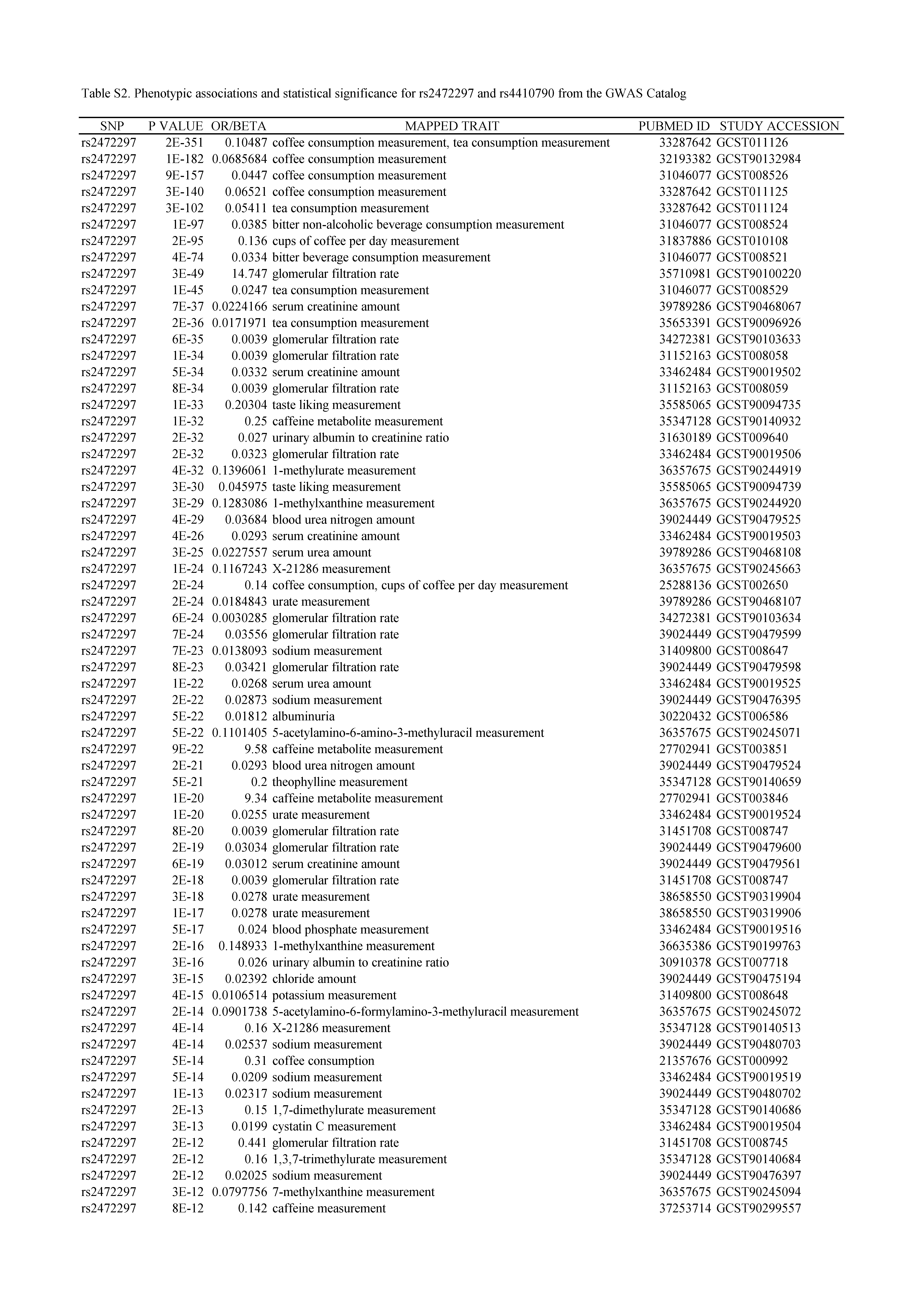


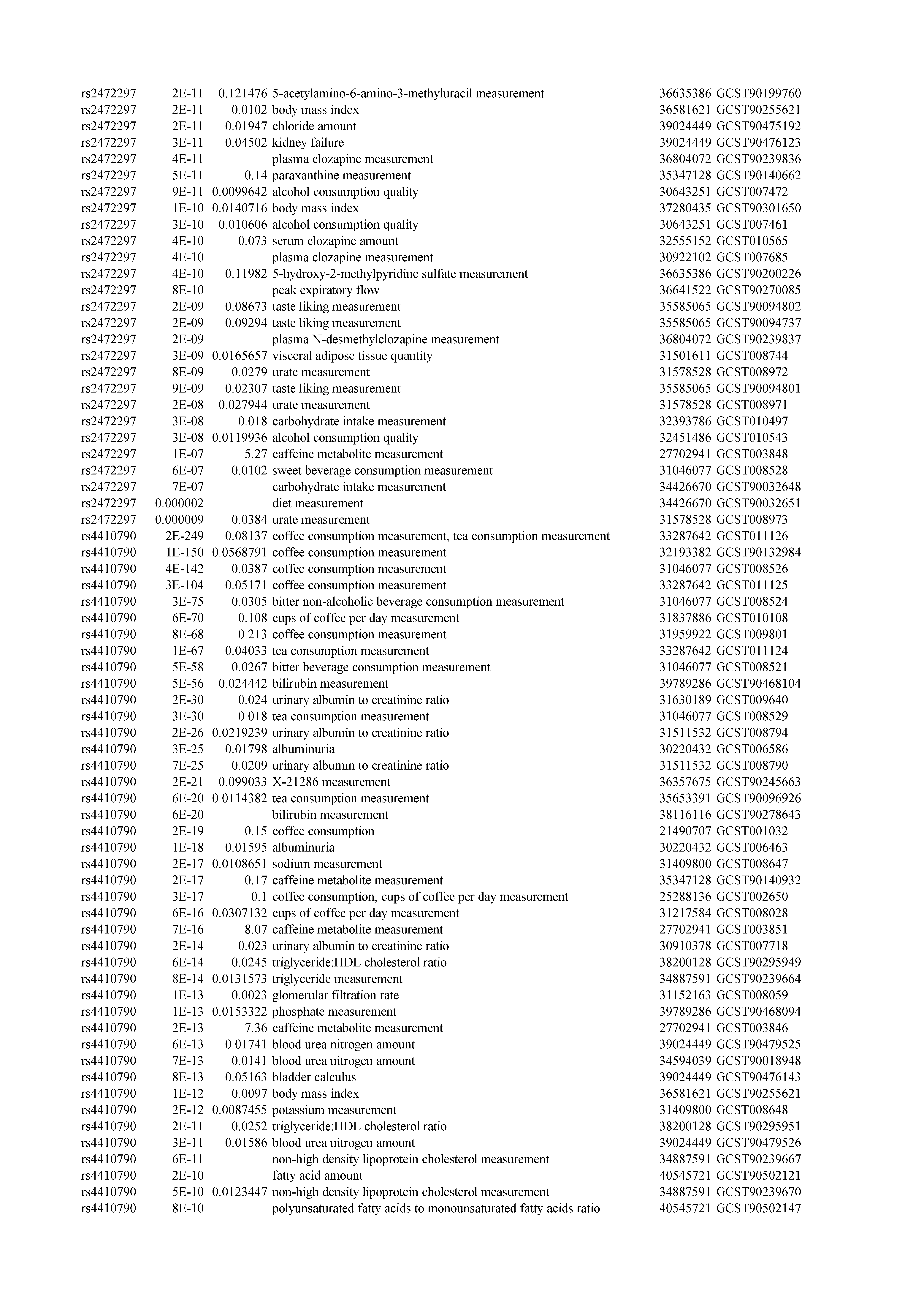


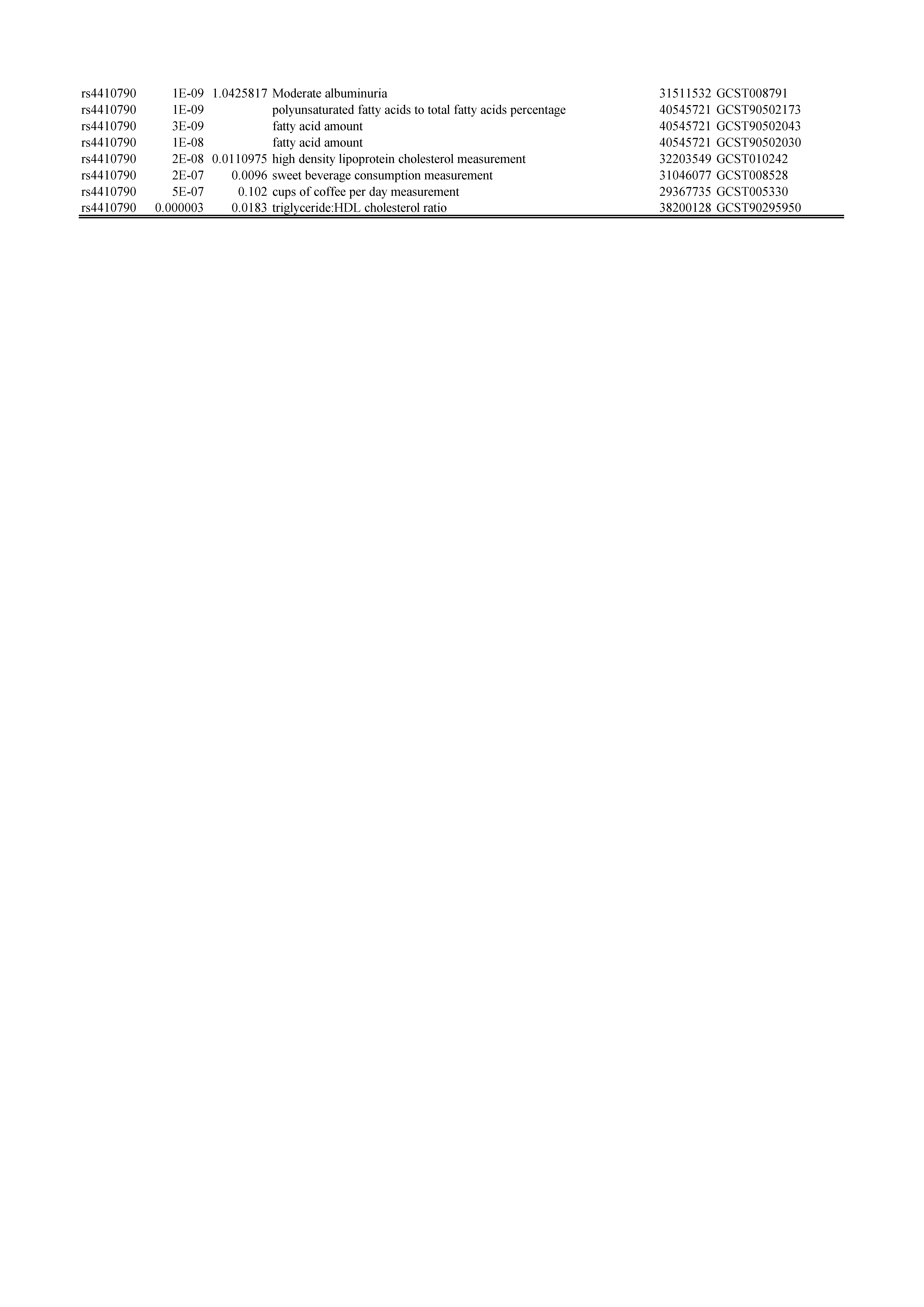


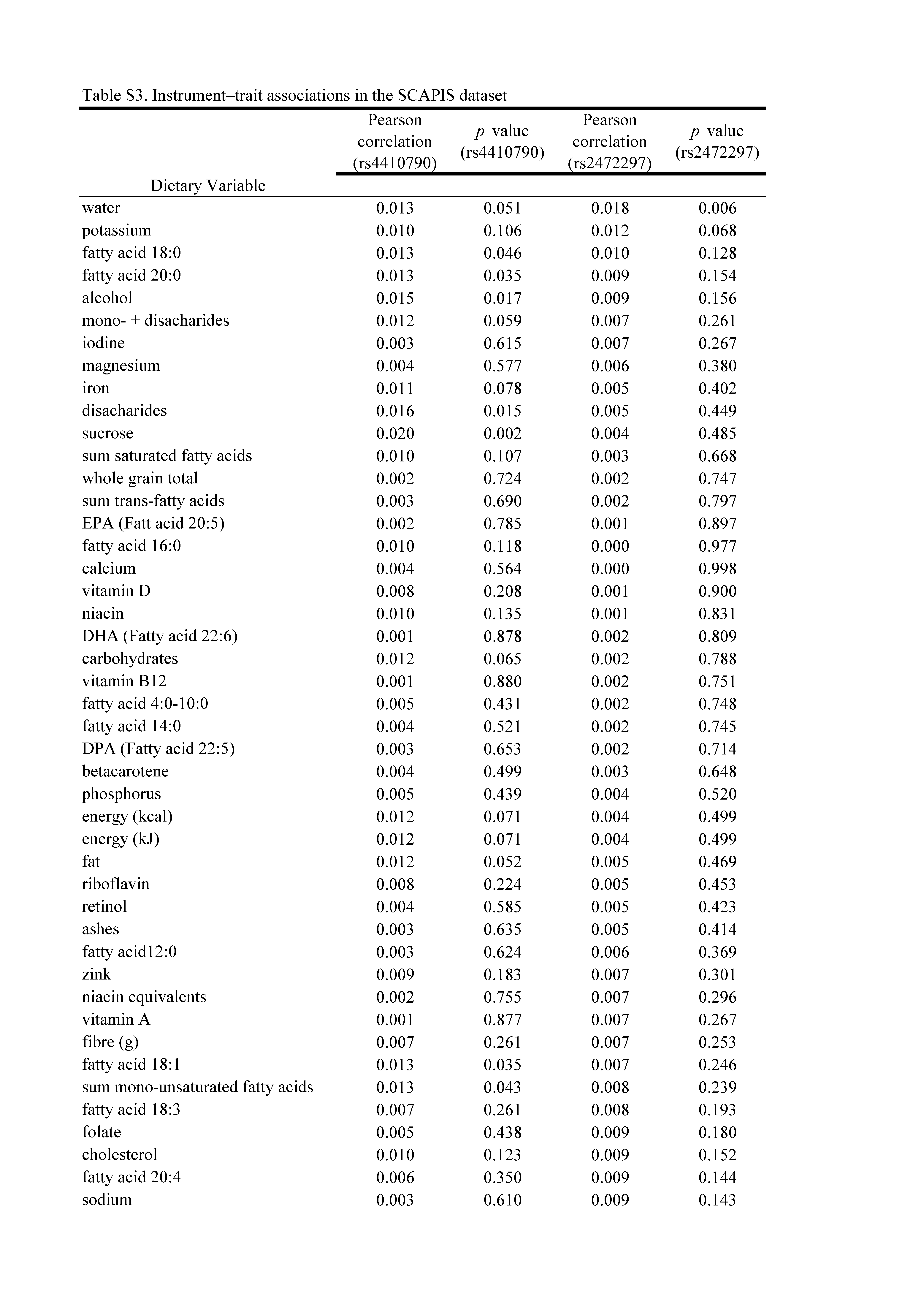


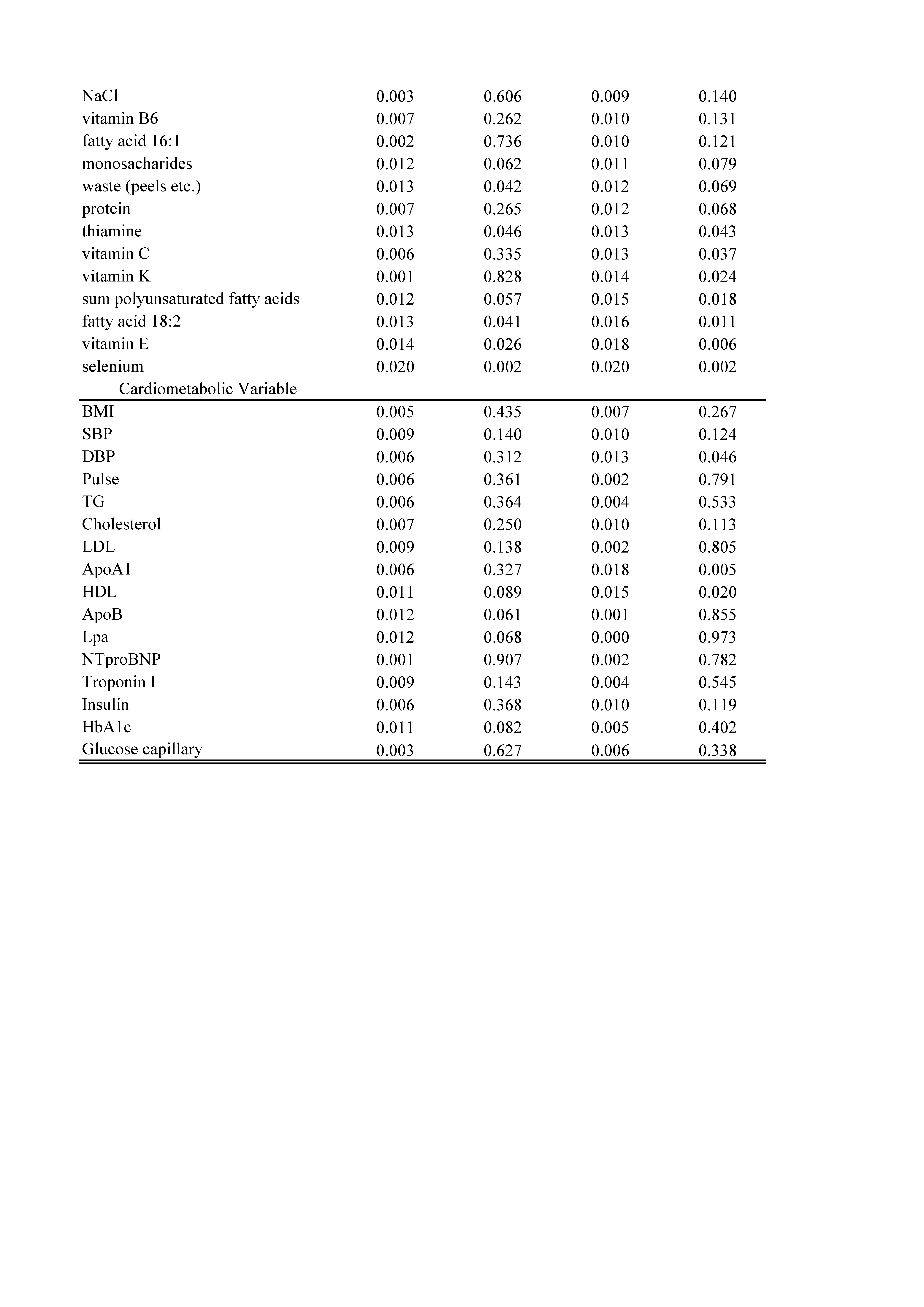


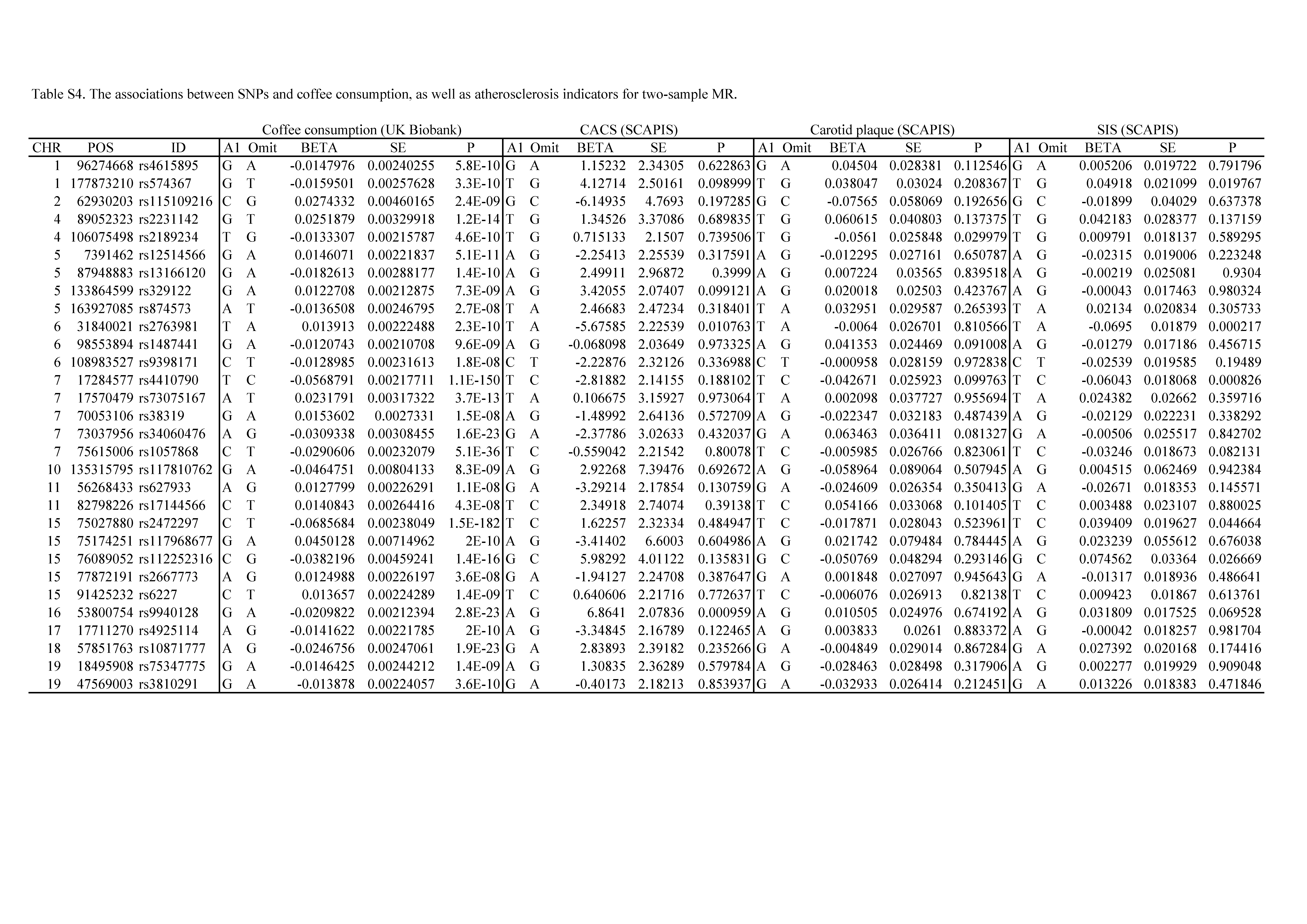


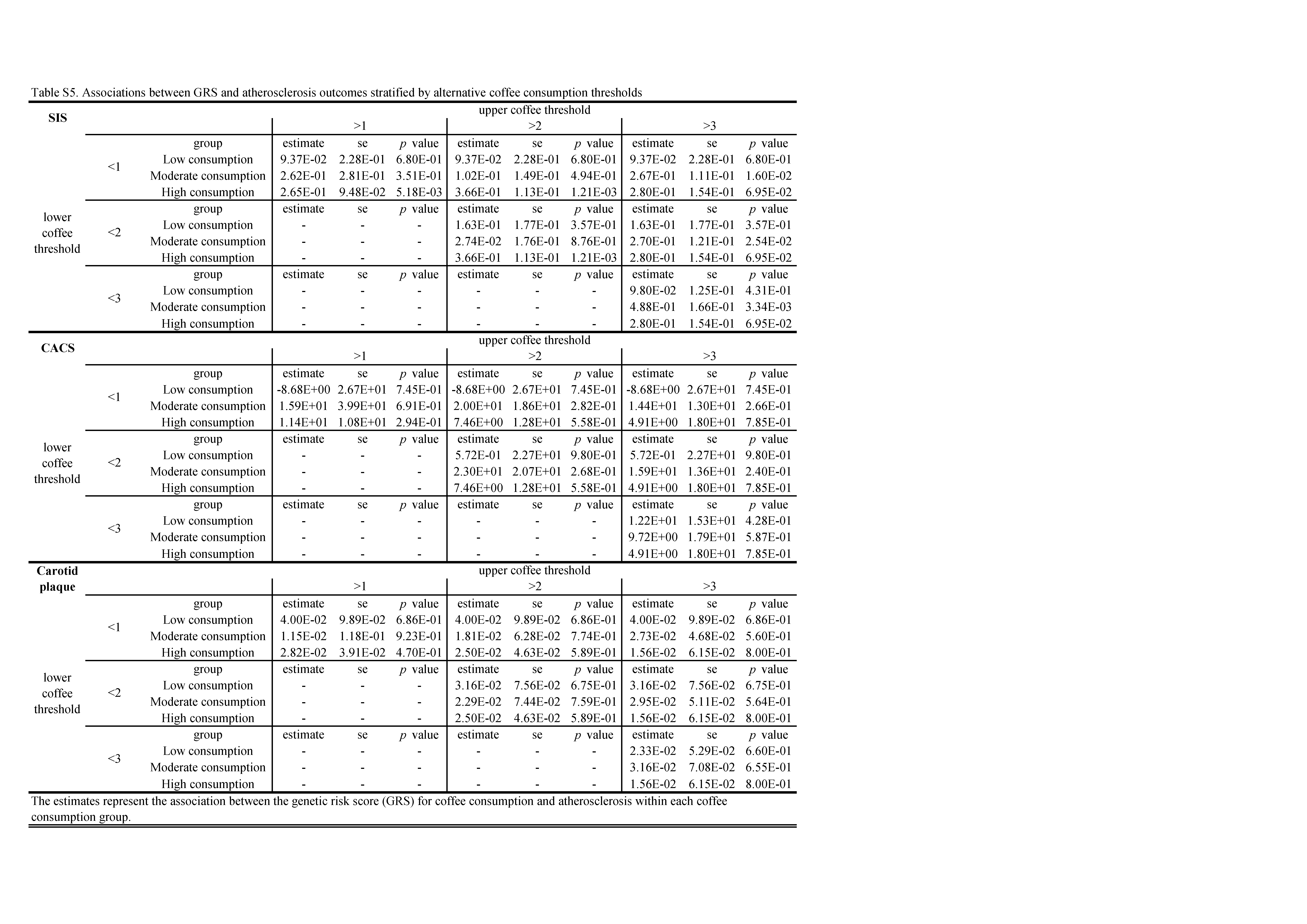


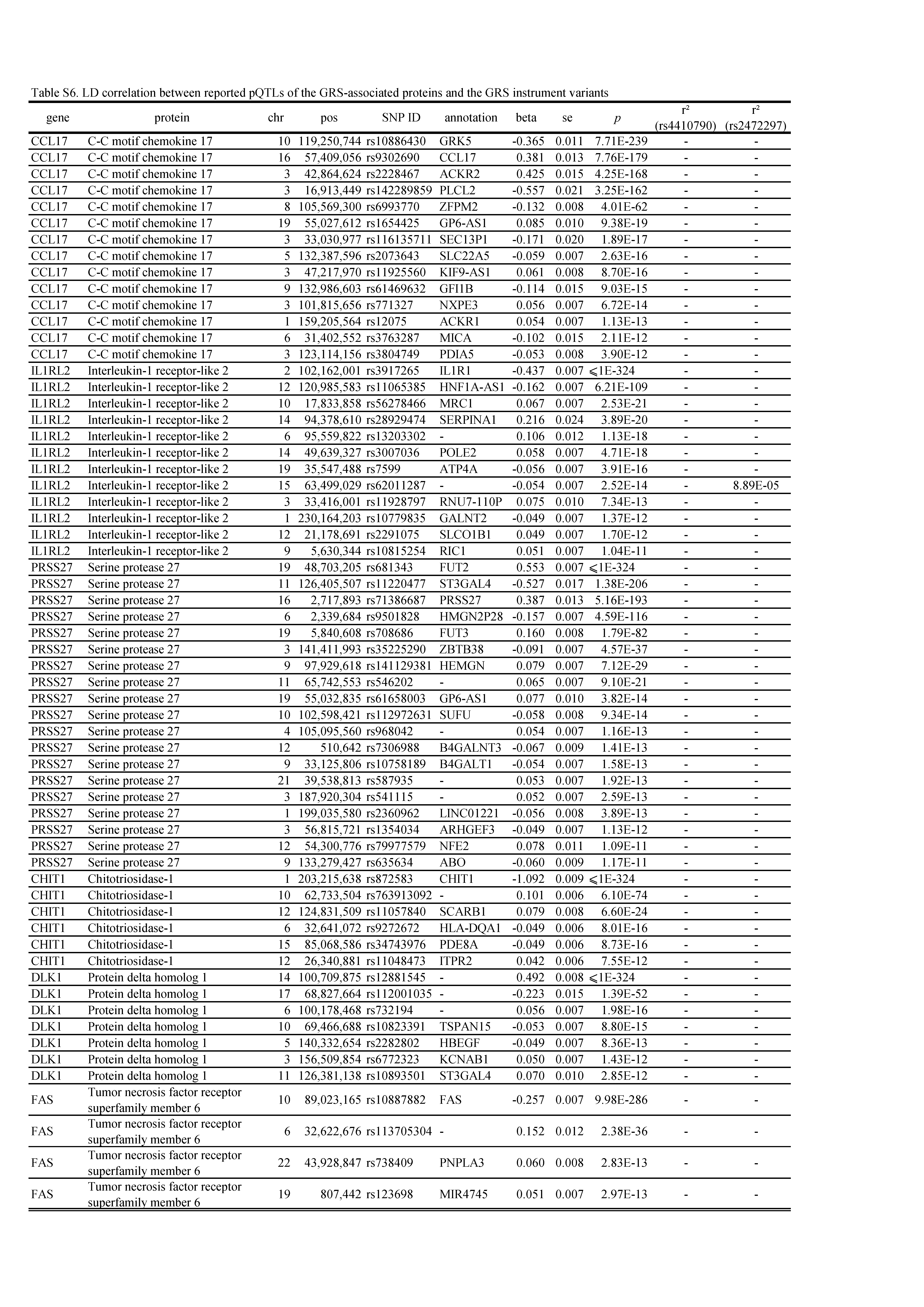


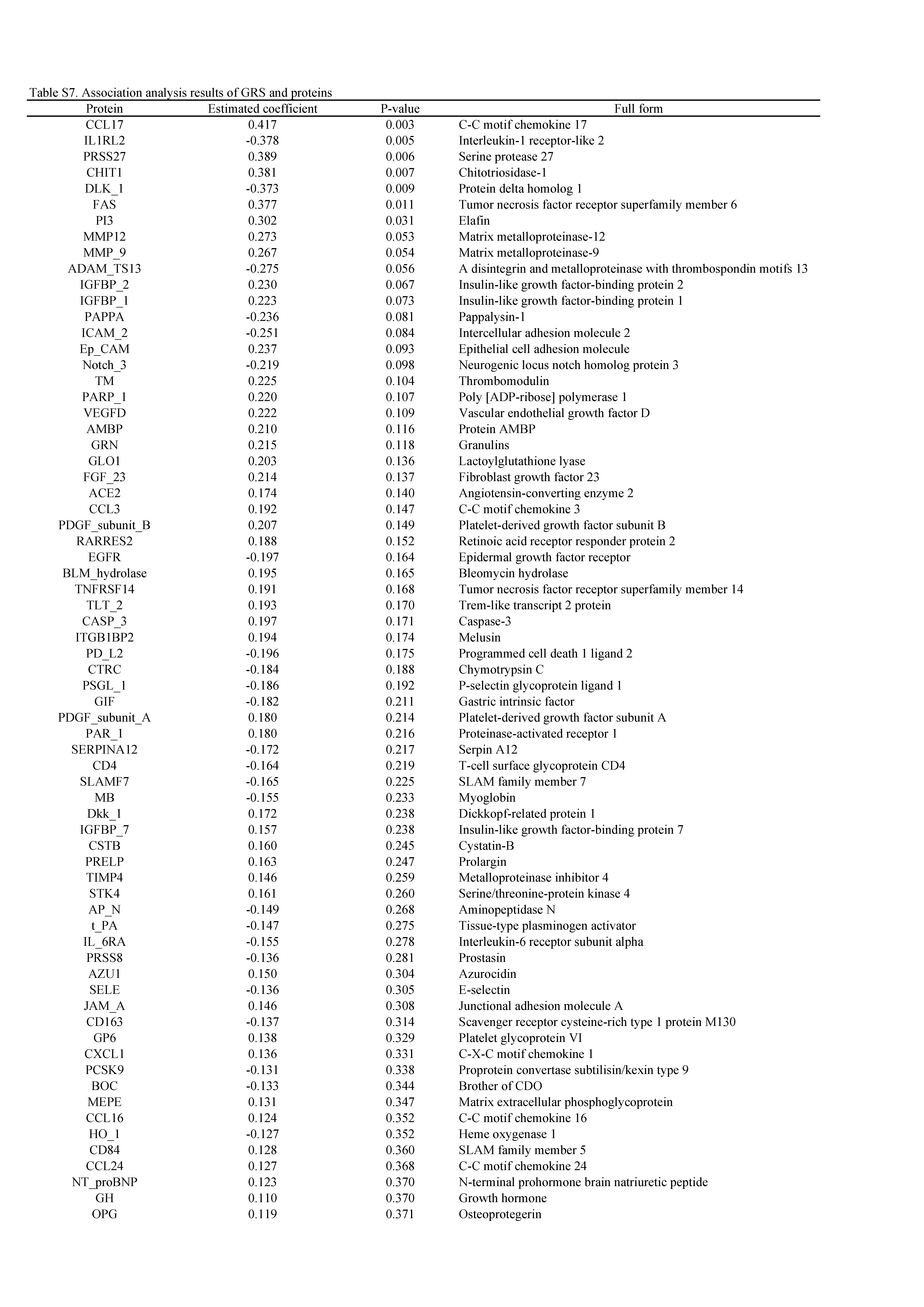


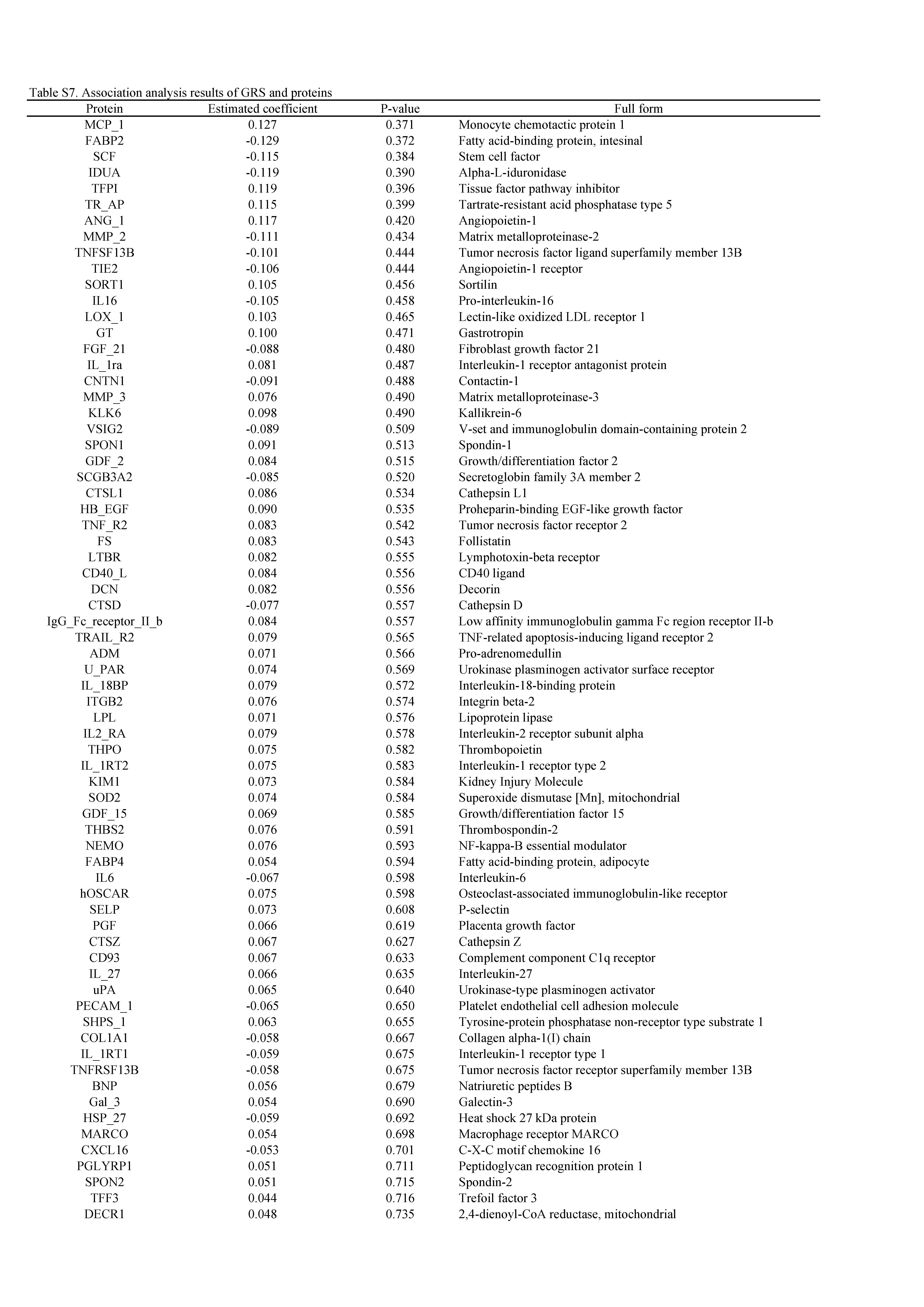

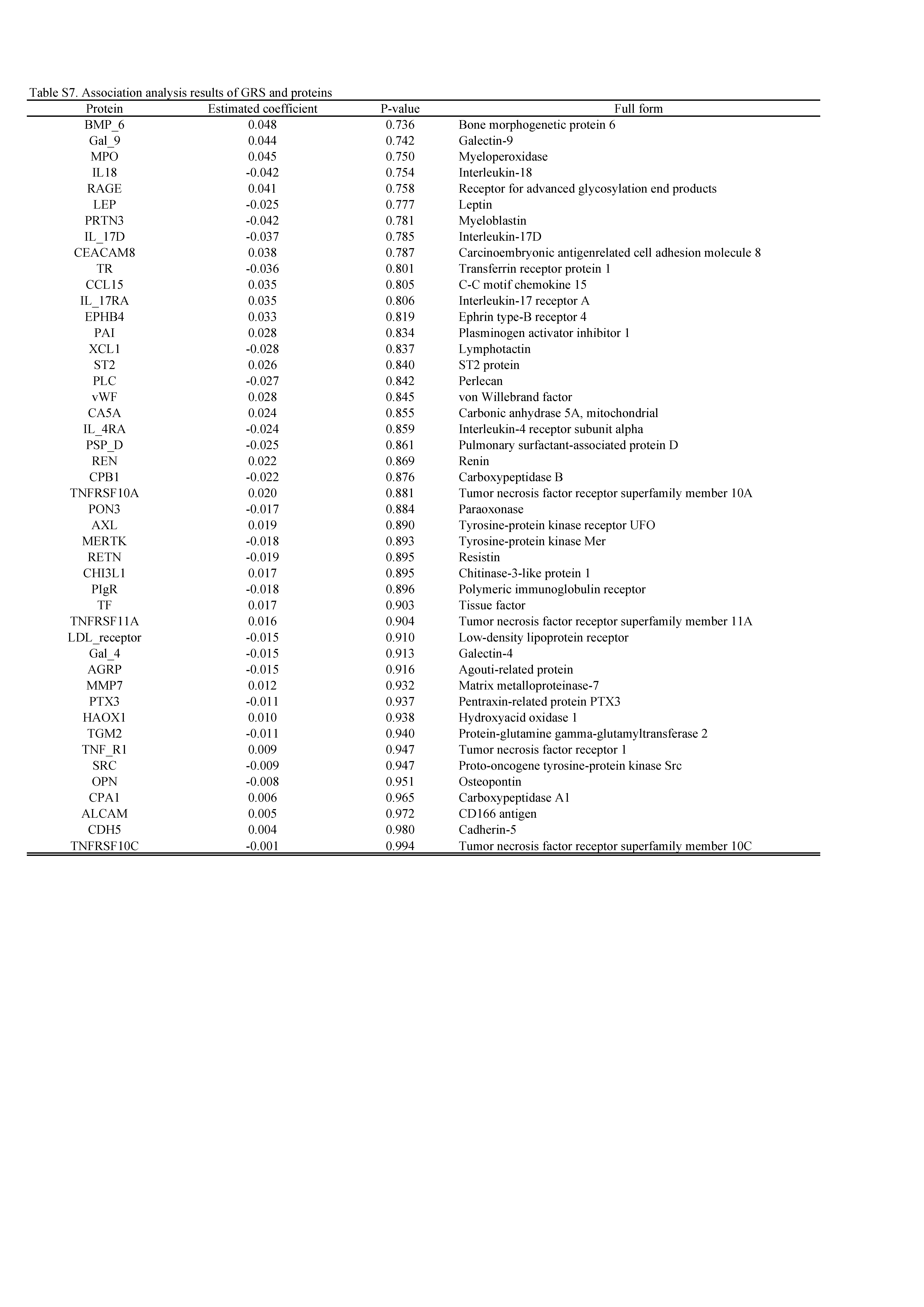


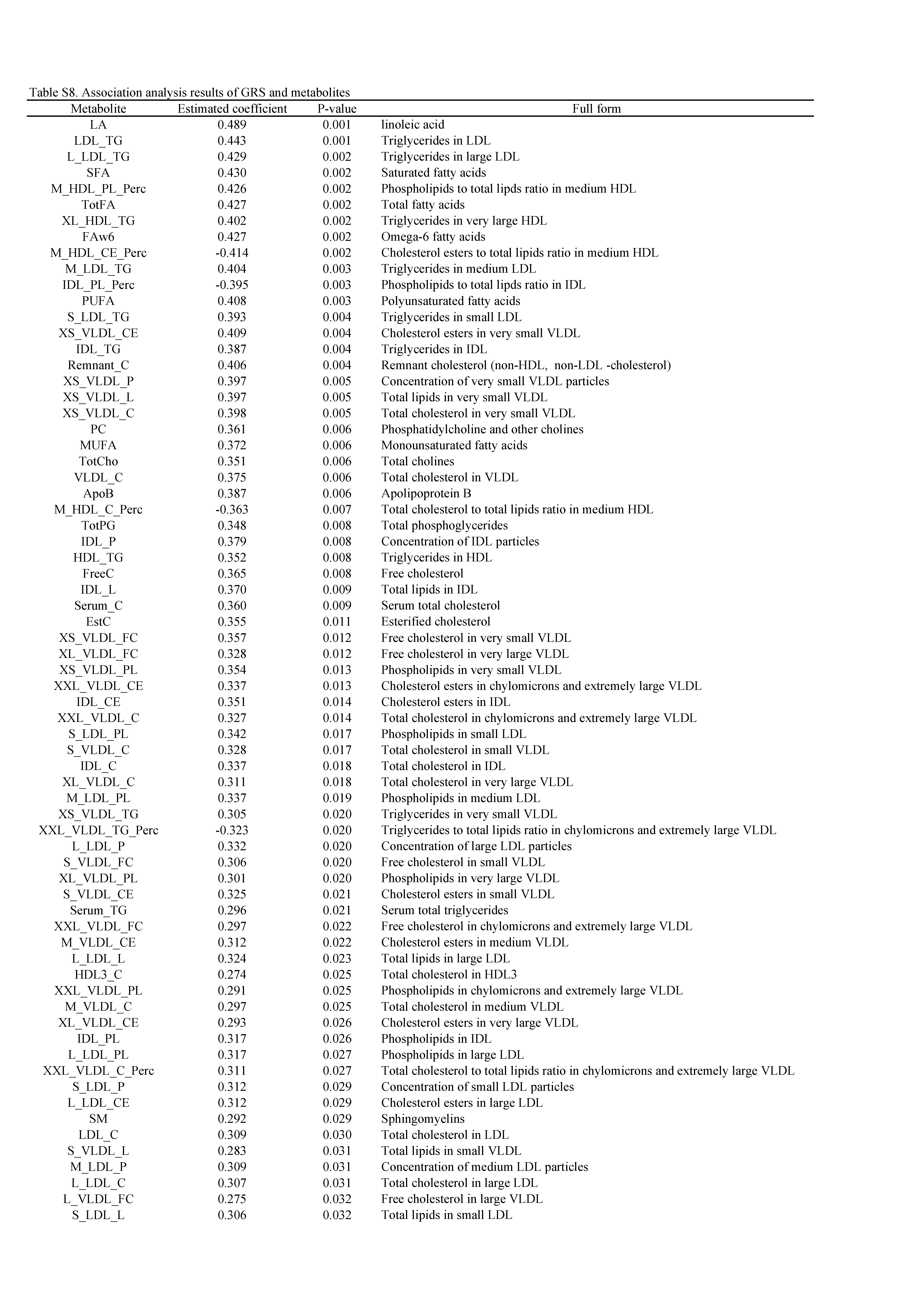


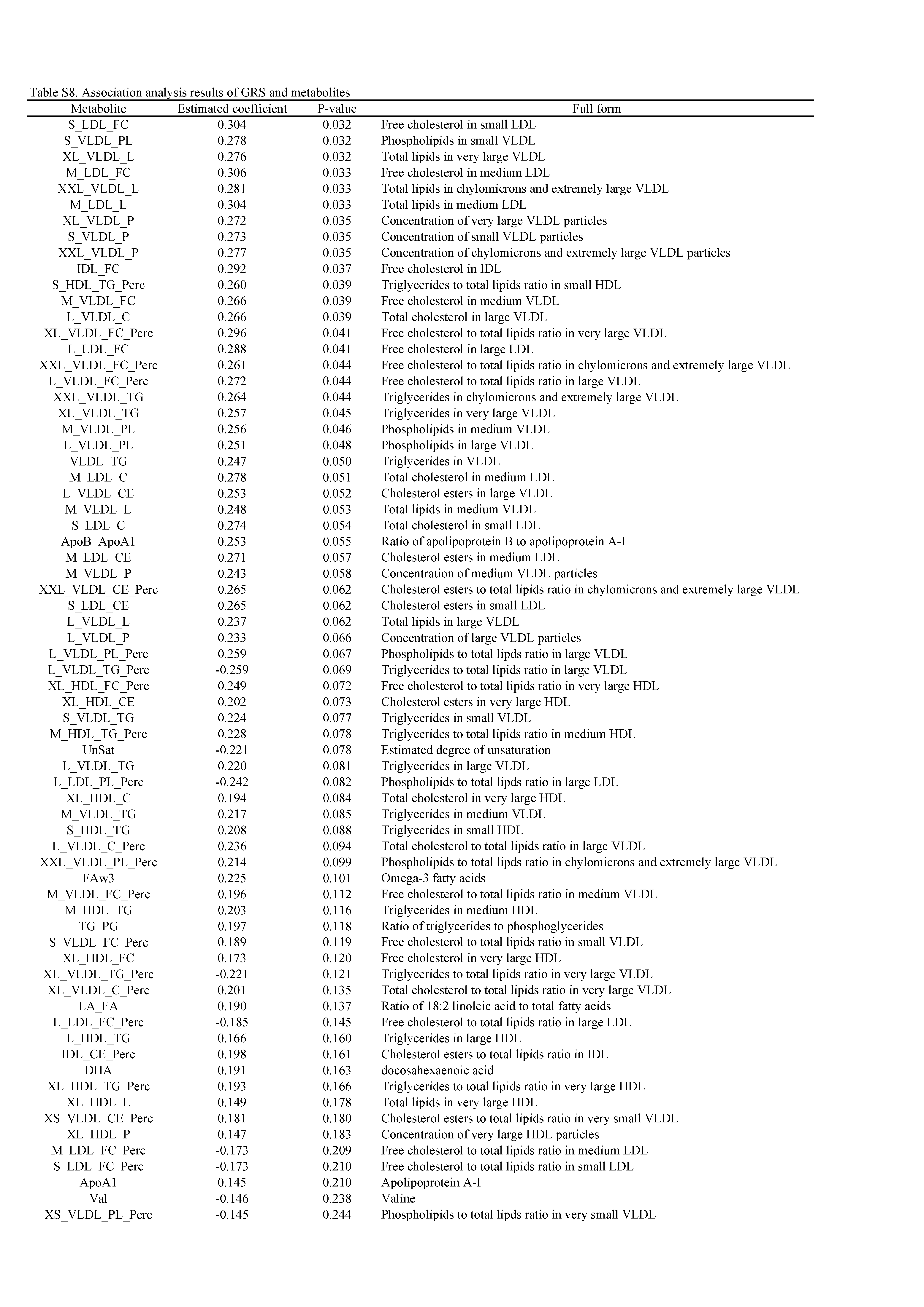


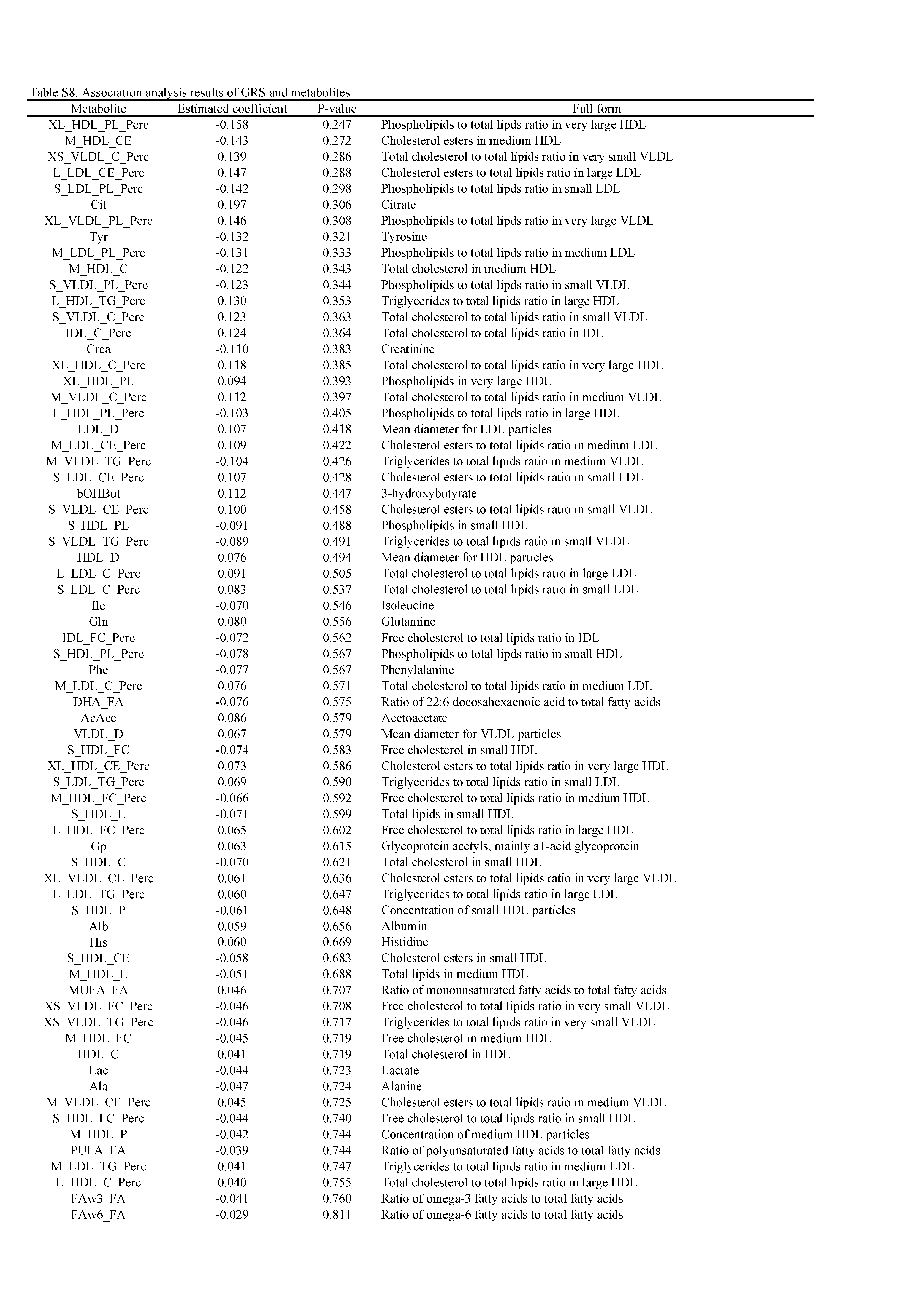


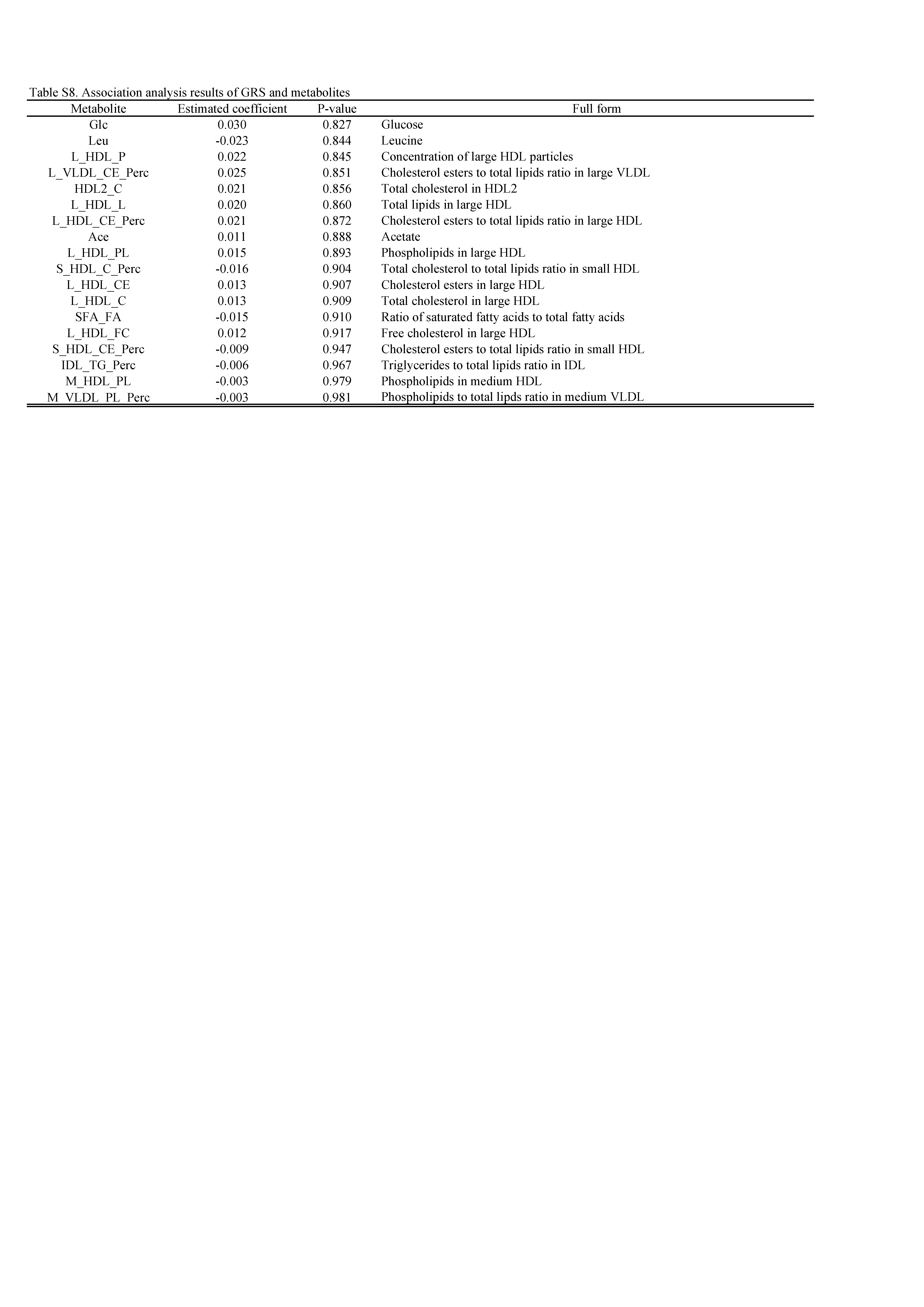

Supplement: Supplementary file 1 — Supplementary Material 1 [file 41598_2026_44122_MOESM1_ESM.docx]
